# Silhouette Scores for Arbitrary Defined Groups in Gene Expression Data and Insights into Differential Expression Results

**DOI:** 10.1186/s12575-018-0067-8

**Published:** 2018-03-01

**Authors:** Shitao Zhao, Jianqiang Sun, Kentaro Shimizu, Koji Kadota

**Affiliations:** Graduate School of Agricultural and Life Sciences, The University of Tokyo, 1-1-1 Yayoi, Bunkyo-ku, Tokyo, 113-8657 Japan

**Keywords:** Hierarchical sample clustering, Bioinformatics, Differential expression analysis, Silhouettes

## Abstract

**Background:**

Hierarchical Sample clustering (HSC) is widely performed to examine associations within expression data obtained from microarrays and RNA sequencing (RNA-seq). Researchers have investigated the HSC results with several possible criteria for grouping (e.g., sex, age, and disease types). However, the evaluation of arbitrary defined groups still counts in subjective visual inspection.

**Results:**

To objectively evaluate the degree of separation between groups of interest in the HSC dendrogram, we propose to use *Silhouette* scores. Silhouettes was originally developed as a graphical aid for the validation of data clusters. It provides a measure of how well a sample is classified when it was assigned to a cluster by according to both the tightness of the clusters and the separation between them. It ranges from 1.0 to − 1.0, and a larger value for the average silhouette (AS) over all samples to be analyzed indicates a higher degree of *cluster* separation. The basic idea to use an AS is to replace the term *cluster* by *group* when calculating the scores. We investigated the validity of this score using simulated and real data designed for differential expression (DE) analysis. We found that larger (or smaller) AS values agreed well with both higher (or lower) degrees of separation between different groups and higher percentages of differentially expressed genes (*P*_DEG_). We also found that the AS values were generally independent on the number of replicates (*N*_rep_). Although the *P*_DEG_ values depended on *N*_rep_, we confirmed that both AS and *P*_DEG_ values were close to zero when samples in the data showed an intermingled nature between the groups in the HSC dendrogram.

**Conclusion:**

Silhouettes is useful for exploring data with predefined group labels. It would help provide both an objective evaluation of HSC dendrograms and insights into the DE results with regard to the compared groups.

**Electronic supplementary material:**

The online version of this article (10.1186/s12575-018-0067-8) contains supplementary material, which is available to authorized users.

## Background

High-throughput technologies, including microarrays and RNA-seq, are widely used to monitor genome-wide expression levels in samples of interest and to compare expression patterns in different groups or conditions (e.g., healthy vs. tumor tissue samples) [1–6]. The latter, comparative analyses are often termed differential expression (DE) analyses and the identification of differentially expressed genes or transcripts (DEGs) is a common approach in studies of the molecular basis of traits [7, 8]. RNA-seq is now the main method used to obtain expression data, but microarrays have provided important insights (e.g., [9]). A main difference between the two technologies is the nature of the expression data: microarrays yield continuous signal intensities, while RNA-seq data provides discrete counts [10, 11]. To appropriately manipulate these expression data, several specialized models (e.g., the negative binomial (NB) model for RNA-seq count data [12–18]) have been proposed.

Another common approach for expression analyses is sample clustering (SC) based on similarity in expression patterns [19–21]. Utilizing its unsupervised characteristic, SC has been used to (i) detect previously unrecognized subtypes of cancer [22, 23], (ii) detect outliers (i.e., outlying samples) [24], (iii) represent overall similarities in expression among various organs [25, 26], and (iv) perform sanity checks to verify expected clustering patterns [27]. When using this approach, researchers can investigate SC results with several possible criteria for grouping (e.g., sex, age, and disease types). However, the evaluation of arbitrary defined groups still counts in subjective visual inspection. Numerical scores indicating the degree of separation between predefined groups would help in the objective assessment of the SC results.

Some researchers empirically know that an SC result of data designed for DE analysis (say, “DE data”) roughly corresponds to the DE result when the groups for the DE analysis are evaluated with respect to the SC result [8]. If individual groups form distinct sub-clusters, where each sub-cluster consists only of members (or samples) in the particular group, DE analysis using such distinct groups would result in many DEGs. Conversely, if members (or samples) in each sub-cluster originate from multiple groups, no or few DEGs would be expected. However, objective evaluation of the relationship between SC results on DE data and the percentages of DEGs (*P*_DEG_) remains lacking [8].

*Silhouettes* is a graphical aid for the interpretation and validation of cluster analysis [28]. In SC, silhouettes provide a measure of how well a sample is classified when it is assigned to a cluster according to both the tightness of the clusters and the separation between them. Therefore, the silhouette scores are calculated for individual samples. By taking the mean over all samples, the average silhouette (AS) value can be obtained. It ranges from 1.0 to − 1.0: a higher (or lower) AS value indicates higher (or lower) degree of separation between clusters. Silhouettes has been successfully used after clustering as a cluster validity measure [20, 29–31].

In this paper, we propose to use *Silhouette* for the objective evaluation of gene expression data based on arbitrary grouping criteria. Although they are independent of SC, silhouette scores measuring the degrees of separation between groups of interest would enable a more objective discussion about the SC result in terms of the groups. We here focus on single-factor DE data where only one grouping criterion is primarily of interest in relation to the DE result. We evaluated the relationship among SC results, DE results, and AS values, using both simulated and real expression data (RNA-seq and microarrays). We found silhouettes (i.e., AS values) to provide a relevant measure for the degrees of separation between groups of interest in SC results. We also found a positive correlation between AS values and DE results.

## Results

In DE analyses, a gene expression matrix is typically generated, where each row indicates the gene (or derivatives), each column indicates the sample, and each cell indicates (i) counts for RNA-seq data or (ii) the signal intensity for microarray data. Our previous observation of the positive correlation between SC and DE results [8] was obtained from an RNA-seq dataset (referred to as *Blekhman*, for short) consisting of 20,689 genes × 18 samples (= 3 species × 2 sexes × 3 biological replicates (BRs)) [32]. The analysis was performed using a hierarchical SC (HSC) algorithm and a DE pipeline, both of which are provided in the R/Bioconductor package TCC [33–35]. TCC implements a robust normalization strategy (called DEGES [36]) that uses functions provided in four widely used packages (baySeq [37], edgeR [38, 39], DESeq [40], and DESeq2) [15]. For simplicity and/or the algorithmic advantage [41, 42], we only used TCC for the DE analysis of RNA-seq data. Specifically, we used the default DE pipeline (*iDEGES/edgeR*-*edgeR* in [33] and *EEE*-*E* in [8]). When performing HSC for all input data, we used the clustering function *clusterSample* with default options ("1 – Spearman’s correlation coefficient (*r*)" as a distance estimate and average-linkage agglomeration) in TCC.

Throughout this study, we filtered out genes with zero counts (or signals) in all samples. For HSC analyses, an additional filtering was performed where genes having identical expression patterns were collapsed. Expression data having those *unique* expression patterns were used for calculating distance defined as “1 – Spearman’s *r*.” This filtering procedure was intended to reduce the negative impact of genes with low expression levels when calculating the distance between samples. For example, the Blekhman data yielded 17,886 genes after the zero-count filtering and DE analyses were performed. After *unique* filtering, 16,560 genes were obtained, and HSC was performed using these genes. For simplicity, we focus on two-group comparisons with three replicates for each group, i.e., (A1, A2, A3) vs. (B1, B2, B3), in most cases. In this study, we use the terms *samples* and *replicates* interchangeably. Our primary interest was to investigate the applicability of Silhouette for the objective evaluation of gene expression data based on arbitrary grouping criteria. By using silhouettes (i.e., AS values) as a relevant measure for the group differentiation in the HSC results, we re-evaluated our previous observations (i.e., the positive correlation between HSC and DE results) [8].

### Representative Relationship between HSC and DE Results with AS

**Fig. 1:**
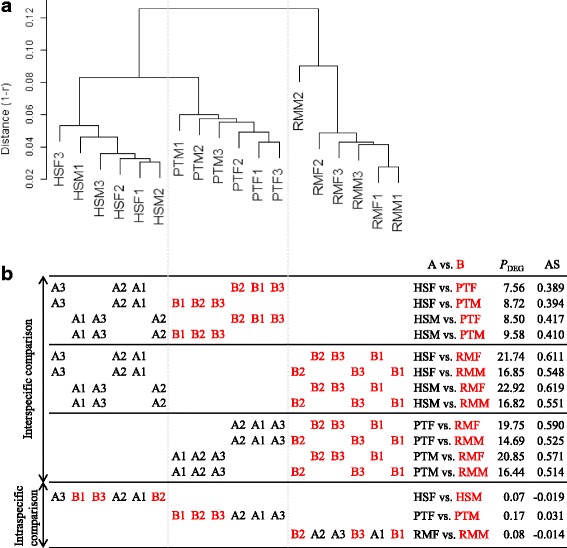
Relationship between the shape of HSC and DE results. **a** HSC dendrogram for Blekhman data consisting of 16,560 genes × 18 samples. The clustering was performed using the “clusterSample” function with default options in TCC. The *unique* filtering (from 17,886 genes to 16,560 genes with *unique* expression patterns across 18 samples) was internally performed in the function to reduce the negative effect on associations in low count regions when calculating Spearman’s *r* as a distance measure. **b** DE results from a total of 15 two-group comparisons with three replicates. The DE pipeline provided in TCC was applied to the Blekhman’s count matrix consisting of 17,886 genes after zero-count filtering. The *P*_DEG_ values and AS values for individual comparisons are provided on the right

We first demonstrate the relationship between HSC and DE results using a representative dataset, the Blekhman data obtained for three species (i.e., the three-group data): humans (HS), chimpanzees (PT), and rhesus macaques (RM) [32]. Briefly, Blekhman et al. studied expression levels in liver samples from three males (M1, M2, and M3) and three females (F1, F2, and F3) from each species/group. Figure 1a shows the HSC dendrogram based on a correlation distance (1 - *r*) metric and average-linkage agglomeration. There were three major clusters, each of which represented a particular species (HS, PT, and RM clusters) and the RM cluster was relatively distant from the other clusters. Different from the clear interspecific discrimination (i.e., high dissimilarity between species), we observed a very low degree of separation between sexes (F vs. M) within each of the three major clusters. That is, samples labelled female (F) and male (M) were intermingled within each species, except for the PTF sub-cluster comprising three female samples (PTF1, PTF2, and PTF3).

Figure 1b shows 15 DE results for two-group comparisons. The percentages of DEGs (*P*_DEG_) satisfying the 10% false discovery rate (FDR) threshold were obtained using TCC with default settings. The four *P*_DEG_ values for the HS vs. PT comparison (7.56–9.58%) were much smaller than those for either the HS vs. RM (16.82–22.92%) or the PT vs. RM comparison (14.69–20.85%). These results are consistent with those of the original study [32] and can primarily be explained by the interspecific distances shown in Fig. 1a. Different from the interspecific comparisons, sex comparisons (F vs. M) showed extremely low *P*_DEG_ values (0.07–0.17%). This is consistent with the lack of separation between female and male samples within each species in the HSC analysis (Fig. 1a).

Silhouette [28] has been successfully employed to estimate the appropriate number of clusters for gene expression data [20, 29–31]. Although Silhouette is generally used for the validation of clustering results, we here employ it independently from clustering. Technically, the term *cluster* is replaced with *group* in the silhouette calculation procedure. For each sample *i*, let *u*_*i*_ be the average distance between *i* and all other samples within the same *group* (e.g., group A). Let *v*_*i*_ be the average distance between *i* and the other *group* (e.g., group B), of which *i* is not a sample member. The silhouette index *s*_*i*_ for sample *i* is calculated as (*v*_*i*_ - *u*_*i*_)/max(*u*_*i*_, *v*_*i*_). The index *s*_*i*_ ranges from − 1 to 1; it is positive if *u*_*i*_ < *v*_*i*_, zero if *u*_*i*_ = *v*_*i*_, and negative if *u*_*i*_ > *v*_*i*_. A larger *s*_*i*_ value indicates increased *group* separation and vice versa. By taking the mean *s*_*i*_ over all samples, the average silhouette (AS) value for each comparison can be obtained (Additional file 1a; right hand in Fig. 1b). The potential applicability of the silhouette unrelated to clustering has been described in the original study [28]. However, to the best of our knowledge, the current study is the first practical application of the concept to estimate the degree of separation between *groups* (not *clusters*) using gene expression data.

It is noteworthy that, in the eight RM-related inter-group comparisons, both *P*_DEG_ and AS values obtained from four RMF-related comparisons were consistently larger than those from the four RMM-related comparisons. For example, for the HSF vs. RMF comparison, *P*_DEG_ = 21.74% and AS = 0.611, while for the HSF vs. RMM comparison, *P*_DEG_ = 16.85% and AS = 0.548. This difference is primarily explained by the smaller average distance of samples in RMF (0.0475) than in RMM (0.0722). Small *P*_DEG_ values (0.07–0.17%) obtained for the sex (i.e., intra-group) comparisons can be explained by the similarity between inter-group distances and intra-group distances. In other words, two-group comparisons showing AS ≈ 0 would result in few, if any, DEGs. The numbers of DEGs (or *P*_DEG_ values) can, of course, vary with FDR thresholds and generally increase when the threshold is less restrictive.

Nevertheless, we confirmed that the general trends for the 15 two-group comparisons were the same at 1%, 5%, 10%, 20%, 30%, and 40% FDR thresholds (Additional file 1b). Based on the definition of FDR, an increase in the *P*_DEG_ value by loosening the FDR threshold does not necessarily indicate an increase in the *true* number of DEGs. For example, *P*_DEG_ = 0.78% at a 40% FDR for the PTF vs. PTM comparison indicates that 0.78 × 0.4 = 0.31% are non-DEGs, and the remaining 0.78 × (1.0–0.4) = 0.47% are, at least statistically, true DEGs. In our experience, the percentage of true DEGs (say *P*_trueDEG_) generally approaches a constant value at a non-stringent FDR threshold, such as 30% or 40%. In this case, the maximum *P*_trueDEG_ value for any sex comparison was ~ 0.5% (Additional file 1c). These results indicate that differences in *P*_DEG_ values with respect to the FDR threshold are not important.

Based on our visual evaluation, the AS values effectively represented the overall relationship between groups of interest in the HSC analysis (shown in Fig. 1a). We think the expressive power in cases of few or no DEGs in the dataset (i.e., AS ≈ 0) is practically promising, but increasing the correlation between *P*_DEG_ (or *P*_trueDEG_) and AS is not practical. This is simply because the *P*_DEG_ value tends to increase as the number of replicates (*N*_rep_) increases [43], suggesting that the correlation is influenced by *N*_rep_.

### Effects of the Number of Replicates (*N*_rep_) on Parameter Estimates

We next investigated the effects of *N*_rep_ on *P*_DEG_ and AS values, using both simulated and real RNA-seq data. The simulated data were constructed as follows: two-group comparison (A vs. B) with 40 replicates per group (*N*_rep_ = 40), 10,000 total genes, of which 20% were DEGs (2000 DEGs and 8000 non-DEGs; *P*_simDEG_ = 20%), the levels of DE were four-fold in individual groups, and the proportions of DEGs up-regulated in individual groups were the same (i.e., 1000 DEGs are up-regulated in group A). For a total of 80 samples (A1, A2, …, A40, B1, B2, …, B40), we obtained *P*_DEG_ = 21.0% at a 10% FDR threshold, AS = 0.2409, and area under the ROC curve (AUC) = 0.9986. The AUC is a widely used measure of both the sensitivity and specificity of the DE pipeline [7, 8, 33, 36]. The value (ranging from 0 to 1) can also be regarded as an overall indicator of the ability to distinguish true DEGs from non-DEGs. A larger AUC value indicates better DE separation and vice versa. The AUC value of 0.9986 indicates nearly perfect separation and the estimated *P*_DEG_ value (21.0% at FDR = 0.1) is in good agreement with the true value (i.e., 20% DEGs or *P*_simDEG_ = 20%).

**Fig. 2:**
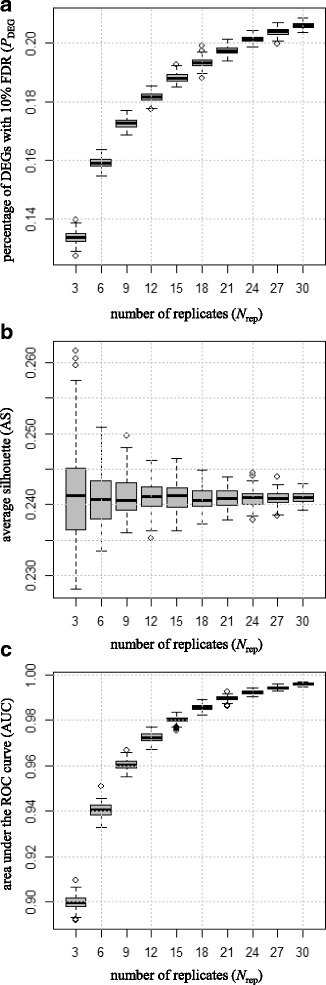
Effects of *N*_rep_ on parameter estimates (simulated data). Bootstrapping results (100 iterations) from simulated RNA-seq data consisting of 10,000 genes × 80 samples with *P*_simDEG_ = 20% are shown. Vertical axes for the boxplots indicate: (**a**) *P*_DEG_, (**b**) AS, and (**c**) AUC values. Horizontal axes indicate the *N*_rep_ values (3, 6, …, 30). It can be seen that *P*_DEG_ and AUC values increase as a function of *N*_rep_, but AS values do not

The DE pipeline was used to examine subsets from the *baseline* matrix with 40 replicates per group (*N*_rep_ = 40). Bootstrap resampling was performed 100 times at *N*_rep_ = 3, 6, …, and 30 (without replacement). Consistent the previous observations [43], the average *P*_DEG_ values increased as a function of *N*_rep_ (Fig. 2a). However, such an increasing trend was not observed for AS (Fig. 2b). This result indicates that the the silhouette (i.e., AS) is independent of *N*_rep_. Note that the *P*_DEG_ value approached to the true value (*P*_simDEG_ = 20%) as *N*_rep_ increased (Fig. 2a). In general, the DE pipeline does not necessarily produce a well-ranked gene list in which true DEGs are top-ranked and non-DEGs are bottom ranked. Given the increase in AUC values in conjunction with increases in *P*_DEG_ (Fig. 2c), this interpretation can be trusted in this case.

Next, the effects of *N*_rep_ under different *P*_simDEG_ conditions (*P*_simDEG_ = 10%, 5%, 2%, 1%, 0.5%, 0.1%, and 0.02%) were investigated. We confirmed that *P*_DEG_, but not on AS, is dependent on *N*_rep_ (Additional file 2). Different from the condition shown in Fig. 2 (*P*_simDEG_ = 20%), however, we observed a transition in the distribution of *P*_DEG_ values at around *P*_simDEG_ = 1%. Although the *P*_DEG_ value monotonously increased as *N*_rep_ increases when *P*_simDEG_ was 20% or more, the *P*_DEG_ value switched to a monotonously decreasing trend when *P*_simDEG_ was 0.1% or less. Overall, the *P*_DEG_ values approached the true values (i.e., the *P*_simDEG_ values) as *N*_rep_ increased. These results indicate that more accurate DE results can be obtained as *N*_rep_ increases, irrespective of the true percentages of DEGs in the data.

A similar analysis was performed using another real RNA-seq dataset consisting of 7126 genes × 96 samples [43, 44]. Ten outlier samples were rejected, following the original study [43], and subsequent zero-count filtering of the original data yielded 6885 genes × 86 samples (*unique* filtering did not have any effect for this dataset). For the data (called *Schurch* for short) comparing two groups (42 wild-type samples vs. 44 Δsnf2 mutant samples), we obtained *P*_DEG_ = 78.1% and AS = 0.7289. Note that the AUC value could not be calculated for the data because, different from simulated data, we do not know which genes are true DEGs. We investigated the effects of *N*_rep_ on parameter estimates. The results were quite similar to those obtained using simulated data (shown in Fig. 2), i.e., *P*_DEG_ was dependent on *N*_rep_, but AS was not (Additional file 3). Note that the distribution of *P*_DEG_ values obtained using TCC (Additional file 3a) was also similar to that obtained using edgeR [39] (Fig. 1a in [43]). This is quite reasonable because the DE pipeline implemented in TCC can be viewed as an iterative edgeR pipeline [8].

### Relationships between *P*_DEG_ and AS Values

**Fig. 3:**
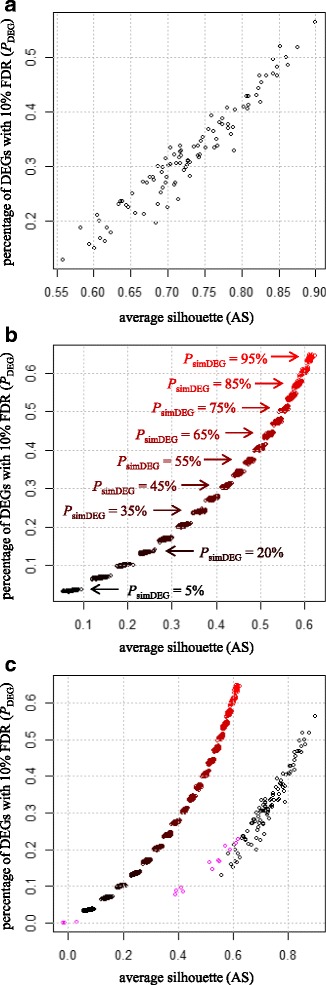
Relationship between *P*_DEG_ and AS values. Scatter plots of *P*_DEG_ vs. AS at *N*_rep_ = 3 are shown. (**a**) Schurch data. The scatter plot shows a detailed relationship between *P*_DEG_ and AS values for Schurch data at *N*_rep_ = 3 (Additional file 3a and 3b). (**b**) simulated data under *P*_simDEG_ = 5%, …, 95%. The scatter plot for *P*_simDEG_ = 20% corresponds to the *P*_DEG_ (ranging from 0.1273 and 0.1397) and AS values (ranging from 0.2281 and 0.2617) for *N*_rep_ = 3 shown in Fig. 2b. (**c**) the results for the mixture as well as the Blekhman data including 15 two-group comparisons shown in Fig. 1b (magenta)

Next, we investigated the relationships between *P*_DEG_ and AS values under a fixed *N*_rep_ of 3. Figure 3 shows the results for (a) Schurch, (b) simulated, and (c) the mixture. For simulated data, we examined 19 *P*_simDEG_ conditions from 5% (black in Fig. 3b) to 0.95 (red in Fig. 3b). Overall, there was a strong positive correlation between *P*_DEG_ and AS values in this condition (Fig. 3c). However, the accurate estimation of *P*_DEG_ using AS is not realistic and accordingly is not a goal of the current study. This is mainly because *P*_DEG_ increases as a function of *N*_rep_, while AS does not (Fig. 2; Additional file 3). In other words, the regression coefficients depend on *N*_rep_. Most importantly, if one wants to calculate *P*_DEG_, there is no need to estimate the AS value; rather, it is only necessary to directly execute the DE pipeline. Nevertheless, as *P*_DEG_ approaches 0, AS also approaches 0. This suggests that *P*_DEG_ values near 0 can be interpreted as a mathematical explanation for AS near 0, i.e., the samples in the two groups (A vs. B) were completely mixed. In statistical terms, this situation is essentially the same as the null hypothesis (*H*_*0*_: A = B). The acceptance of *H*_*0*_ (AS = 0) indicates there are no or few DEGs in the two-group data (*P*_DEG_ = 0). In this sense, AS could be used as helpful information for the interpretation of DE results, especially when only a few statistically significant DEGs are obtained.

It should be noted that the distribution shown in Fig. 3c (right panel) differs substantially from the distribution for real data (Blekhman [32] and Schurch [43]) and simulated data, but the *shapes* of the distributions were similar. For example, the *P*_DEG_ value at AS = 0.6 was approximately 0.6 for the simulated data, while *P*_DEG_ for real data was approximately 0.2. Since the AS value for the simulated data at *P*_DEG_ = 0.2 was approximately 0.3, the difference for AS at *P*_DEG_ = 0.2 was 0.3. Similarly, the difference for *P*_DEG_ at AS = 0.6 was 0.4. It should also be noted that the distribution of values for Blekhman (magenta) and Schurch (black with AS > 0.5) was different (Fig. 3c). While low AS values (− 0.019–0.619) and low *P*_DEG_ values (0.07–22.92%) were obtained for the Blekhman data, high AS values (0.5585–0.8998) and high *P*_DEG_ values (13.03–56.34%) were obtained for the Schurch data. The difference can be explained by the intra-group distances. For the Schurch data, including 42 wild-type samples (group A) and 44 Δsnf2 mutant samples (group B), the distances for groups A and B were 0.0144 and 0.0084, respectively. The values obtained for the Schurch data were clearly smaller than those obtained for the Blekhman data (> 0.04; Fig. 1a). According to a previous study [43], the Schurch data represents a best-case scenario for DE pipelines, since the within-group biological variation (BV) is low. As the BVs roughly correspond to the intra-group distances, many other real RNA-seq data may display low *P*_DEG_ and AS values compared to those obtained for the Schurch data.

### Analyses of two Additional Real RNA-Seq Datasets

We further investigated two other real RNA-seq datasets available at the ReCount website [45]. The first dataset (called Bottomly [46]) consisted of 36,536 genes × 21 samples. Briefly, Bottomly et al. studied the expression levels of two common inbred mouse strains used in neuroscience research, i.e., 10 C57BL/6J strains (A1, A2 …, A10) and 11 DBA/2J strains (B1, B2, …, B11). DE analyses (i.e., estimates of *P*_DEG_ values) were performed using 13,932 genes after zero-count filtering. AS calculations and HSC were performed using 13,133 genes after *unique* filtering. The results for this dataset comparing 10 vs. 11 samples were *P*_DEG_ = 11.0% at a 10% FDR threshold and AS = 0.1872. Regarding the effects of *N*_rep,_ we observed similar trends to those obtained using the Schurch data (Fig. 3a), i.e., *P*_DEG_ increased as a function of *N*_rep_, while AS did not (Additional file 4a–b). Despite similar trends, the values obtained for the Bottomly data were clearly lower than those for the Schurch data. For example, the Bottomly data (and Schurch data) showed on average *P*_DEG_ = 3.81% (32.4%) and AS = 0.1874 (0.7306) at *N*_rep_ = 3 (Additional files 3 and 4). These findings suggested that the *P*_DEG_ values were lower for Bottomly than for Schurch because the AS value for Bottomly was lower than that of Schurch.

In general, high (or low) AS values indicate clear (or unclear) separation of groups. The HSC dendrogram for the Bottomly data showed relatively unclear separation of two groups (Additional file 4c; AS = 0.1872) compared to the separation of groups for the Schurch data (Additional file 3c; AS = 0.7289). Nevertheless, as also implied by the positive AS value, the degree of inter-group separation for the Bottomly data was not random. For example, by visual inspection, we identified four clusters, each of which consisted only of samples within the same group (Additional file 4c). These clusters primarily explained the estimated *P*_DEG_ values and positive AS values. The highest values for *P*_DEG_ (=24.7%) and AS (= 0.3524) among 100 trials at *N*_rep_ = 3 were obtained when comparing (A3, A4, A6) vs. (B1, B3, B8). This is reasonable because five of the six samples (except for A3) were members of either the *A2* or *B1* cluster. Relatively high values can be obtained by comparing two groups in which all members belong to either the *A2* or *B1* clusters. Indeed, we observed *P*_DEG_ = 34.8% and AS = 0.4623 when comparing (A2, A4, A6) vs. (B1, B2, B3), though this comparison was not included in the 100 original trials shown in Additional file 4a–b.

Different from the Schurch data where the impact of sampling effects shrunk as *N*_rep_ increased (Additional file 3a), we did not observe shrinkage for the Bottomly data around *N*_rep_ = 3–7 (Additional file 4a). This can also be explained by the four clusters mentioned above. For example, the highest values for *P*_DEG_ (=23.5%) and AS (= 0.2699) among 100 trials at *N*_rep_ = 6 were obtained when comparing (A2, A3, A4, A6, A7, A9) vs. (B1, B2, B3, B4, B8, B10). All eight samples in the *A2* and *B1* clusters was included in the comparison. Additionally, a comparison between the two clusters, i.e., (A2, A4, A6, A7) vs. (B1, B2, B3, B8), yielded *P*_DEG_ = 31.8% and AS = 0.3701. Accordingly, the decreases in *P*_DEG_ (=31.8% to 23.5%) and AS (0.3701 to 0.2699) values by the addition of four samples (A3, A9, B4, and B10) not included in the two clusters are reasonable. We observed that the impact of sampling effects tends to shrink as *N*_rep_ (> 7) increases. This is probably because the maximum number of samples in the four clusters is seven for the *B4* cluster; the addition of samples not included in the cluster can contribute to decreases in the *P*_DEG_ and AS values.

The second dataset (called Cheung [47]) consisted of 52,580 genes × 41 samples. Briefly, Cheung et al. studied the expression levels of human B-cells using 17 females (A1, A2, …, A17) and 24 males (B1, B2, …, B24). The DE analyses (i.e., estimates of *P*_DEG_ values) were performed using 12,410 genes after zero-count filtering. AS calculation and HSC were performed using 11,738 genes after *unique* filtering. The results for this dataset comparing 17 vs. 24 samples were *P*_DEG_ = 0.169%, SNR = 1.023, and AS = 0.0118. The values were considerably lower than those obtained for both the Schurch and Bottomly data and were similar to those for the three sex comparisons (Fig. 1b). This result is intuitively reasonable, as gene expression levels in B-cells are not expected to differ greatly between females and males.

We did not observe an increasing trend for *P*_DEG_ values as *N*_rep_ increased (Additional file 5a). The average *P*_DEG_ values for 100 trials at *N*_rep_ = 3, 5, 7, 9, 11, 13, and 15 were 0.631%, 0.291%, 0.399%, 0.254%, 0.492%, 0.325%, and 0.219%, respectively. These values as well as the distribution were quite similar to those obtained from simulated data with *P*_simDEG_ = 0.5% (Page 5 in Additional file 2a). This result suggests that the increase of *N*_rep_ does not contribute to the increase of *P*_DEG_ when AS is near 0. Since AS is independent of *N*_rep_, no or few DEGs (*P*_DEG_ < 1%) would be obtained when AS < 0.1 for count data (Additional file 5b). The intermingled nature of the HSC dendrogram for the Cheung data (Additional file 5c) also supports this inference; AS can be utilized to interpret the DE results.

### Analysis of two Microarray Datasets

We finally investigated two microarray datasets obtained using the Affymetrix Rat Genome 230 2.0 Array (GPL1355). The first dataset (called Nakai [4]) consisted of 31,099 probesets (which can be viewed as *genes*) × 24 samples (= 3 tissues × 2 conditions × 4 BRs). Briefly, Nakai et al. studied the expression levels of genes in brown adipose tissues (BAT), white adipose tissues (WAT), and liver tissues (LIV). They compared two conditions (fed vs. fasted for 24 h) for each tissue type. We here denoted the fed BAT samples *BAT_fed*, the 24 h–fasted LIV samples *LIV_fas*, and so on. To quantify expression from the probe-level data (i.e., Affymetrix CEL files), we applied three algorithms (MAS [48], RMA [49], and RobLoxBioC [50]). Different from RNA-seq data represented as integer counts, microarray data are expressed as continuous signals and in most cases are log-transformed. We therefore applied a specialized DE pipeline for microarray data provided in the package limma [51], instead of the DE pipeline used for RNA-seq data in TCC.

As expected based on the nature of microarray expression signals, zero signal values were not obtained for any genes in all samples and all genes displayed *unique* expression patterns. Accordingly, the subsequent analysis of microarray data was performed based on total set of genes (= 31,099). The HSC dendrogram for the Nakai data displayed three major clusters corresponding to the three tissue types (LIV, WAT, and BAT clusters) for all quantification algorithms (MAS, RMA, and RobLoxBioC; Additional file 6a). Since the experimental design and the HSC dendrogram were very similar to those of the Blekhman data (Fig. 1), these microarray data can be regarded as the counterpart.

We performed 15 two-group comparisons with four BRs for each group, i.e., (A1, A2, A3, A4) vs. (B1, B2, B3, B4). Overall, we observed highly similar trends for the Nakai data and the Blekhman data (Additional file 6b). For MAS-quantified data, for example, four *P*_DEG_ values in the BAT vs. WAT comparison (24.49–34.98%) were smaller than those in the BAT vs. LIV comparison (41.79–44.63%) or WAT vs. LIV comparison (39.74–44.05%). Different from the clear inter-tissue differentiation (i.e., high dissimilarity between tissues), we detected a relatively low degree of separation between conditions (fed vs. fasted) within each of the three major clusters. The *P*_DEG_ values for the fed vs. fasted comparison were 4.5–8.79%. Of these three comparisons, the intra-BAT comparison (i.e., BAT_fed vs. BAT_fas) showed the highest *P*_DEG_ (8.79%) and AS (0.207) values.

We observed similar results for RobLoxBioC-quantified data and relatively dissimilar results for RMA-quantified data. In particular, for the RMA-quantified data, we detected higher *P*_DEG_ and AS values compared to those of the other data. There are several potential explanations. RMA treats a batch of arrays simultaneously, while MAS and RobLoxBioC treat each array independently. RMA tends to overestimate sample similarity [52]. Combinations of DE pipelines with different quantification algorithms might also explain the higher *P*_DEG_ values observed in RMA-quantified data: limma is more compatible with MAS than RMA [53, 54]. Nevertheless, we observed a clear positive relationship between *P*_DEG_ and AS values, suggesting that AS is also applicable to microarray data.

The second dataset (called Kamei [55]) consisted of 31,099 *genes* × 10 samples (five BRs per group). Briefly, Kamei et al. compared gene expression in livers for rats fed a low-iron diet (approximately 3 ppm iron) for 3 days and a normal diet (48 ppm iron) as a control. The *P*_DEG_ and AS values obtained (*Iron_def* vs. *Control*) were close to zero and the HSC dendrogram showed an intermingled structure (Additional file 7). These results indicate that the Kamei data can be regarded as a counterpart of the Cheung data (Additional file 5). AS can be utilized as supporting information to interpret DE results for both RNA-seq and microarray data, especially when no or few DEGs were obtained.

We should note that one sample (*Iron_def1*) was a clear outlier in the HSC dendrogram for the RMA-quantified data, but not in the other dendrograms (Additional file 7). *Iron_def3* was the most distant from the other samples in MAS- and RobLoxBioC-quantified data. This difference can also be explained by tendency of RMA to overestimate sample similarity [52]. Indeed, the average distance (0.007) among samples in RMA-quantified data was considerably lower than those for the other datasets (0.043 for MAS and 0.037 for RobLoxBioC). The expression levels for the two microarray datasets (Nakai and Kamei) were obtained using the same device (i.e., the Affymetrix Rat Genome 230 2.0 Array), indicating that the datasets can be directly compared. The average distances among ten liver samples in the Kamei data were clearly lower than those among eight liver samples (LIV) in the Nakai data (0.078 for MAS, 0.022 for RMA, and 0.070 for RobLoxBioC). These results suggest that the differences in the most distant samples in the Kamei data (*Iron_def1* in RMA data and *Iron_def3* in the other data) are within the error range.

HSC dendrograms of the merged data provided several insights (Additional file 8). First, the ten liver samples in Kamei data formed a tight cluster, even after adding the Nakai data, and formed a larger cluster when the eight liver samples from the Nakai data were included, confirming the overall similarities among various tissues (i.e., a sanity check) [25–27]. Second, compared to 24-h fasting, the short-term iron-deficient diet might not result in significant differences in gene expression. This conclusion is supported by adding other publicly available dataset(s) for identical (or highly similar) tissues. It may be more important to add independent, publicly available datasets than to perform more detailed analyses using a single dataset. Third, an appropriate distance measure is important. The distance was defined here as (1 - Spearman’s *r*); this definition is widely used [21, 27]. Since the distance ranges from 0 to 2, the interpretation is relatively easy compared to the interpretation of Euclidean distances, which range from 0 to ∞. We indeed understood the extremely high similarity among the ten liver samples in the Kamei data in the context of the very small distance values. In general, distance information is not interpreted so broadly in HSC analyses, but examinations of both the distance (1 - *r*) and AS may be useful.

## Discussion

In this study, we proposed to use silhouettes (i.e., AS values) as an objective measure for the degrees of separation between groups of interest based on expression data. To our knowledge, the use of AS independent from HSC is the first practical application in the field of expression analysis. Our main findings are (i) AS is an effective indicator of the overall relationship in the HSC dendrogram based on arbitrary grouping criteria; (ii) AS values are independent of *N*_rep_, while *P*_DEG_ values obtained from DE analysis are fundamentally dependent on *N*_rep_; and (iii) there is a positive correlation between AS and *P*_DEG_ values under a fixed *N*_rep_. It is not necessary to estimate *P*_DEG_ from AS values because DE results (including *P*_DEG_) can directly be obtained via the DE pipeline. The AS provides helpful information for interpreting DE results as well as HSC results.

Based on the current results, we conclude that our calculation procedure for AS is appropriate. The procedure consists of 1) filtering genes with low expression, 2) calculating distances among samples, and 3) calculating the AS values based on distance estimates. The high similarity among samples in the Kamei data could be detected by investigating the distances defined as (1 - Spearman’s *r*). Considering this finding in addition to other data, some samples could be misidentified as outliers (e.g., *Iron_def1* in Additional files 7 and 8). In addition to the AS value obtained for the groups of interest, (i) the investigations of distances among samples and/or groups in the dataset and (ii) comparison with other datasets obtained from the same or similar samples are practically important.

Of course, there are true outliers, e.g., ten outlying samples in the original Schurch data [43, 44]. We manually eliminated the ten outliers as determined in the original study [44] and analyzed 86 *clean* samples in this dataset (Fig. 3a; Additional file 3). The values obtained without outliers (*P*_DEG_ = 78.1% and AS = 0.7289) were clearly higher than those with outliers (*P*_DEG_ = 74.7% and AS = 0.6530), indicating the importance of developing methods for the automatic detection of outliers [55, 56]. Our preliminary analysis for the original data using an existing method [57] successfully detected nine of the ten *true* outliers as well as three false positives. We obtained a promising result (*P*_DEG_ = 77.6% and AS = 0.7301) using the remaining 84 samples. Rational removal of outlying samples would yield better DE results. We expect that AS would help objective evaluation of the changes in the DE results accompanying outlier removal.

In practice, Silhouettes can be utilized as supporting information to interpret DE results, especially when no or few DEGs are obtained. As demonstrated by several examples (e.g., Additional file 7), we actually encounter such expression data. Silhouettes enables us to discuss the DE results as well as SC dendrograms more objectively.

## Conclusion

Silhouettes is useful for exploring data with predefined group labels. It would help provide both an objective evaluation of SC dendrograms and insights into the DE results with regard to the compared groups. The use of this measure would enable a more objective discussion about the SC result in terms of the groups.

## Methods

Most of the analyses were performed using R (ver. 3.3.2) [34] and Bioconductor [35]. The versions of major R packages used in the study were TCC ver. 1.14.0, edgeR ver. 3.16.5, ROC ver. 1.50.0, cluster ver. 2.0.5, affy ver. 1.44.0, and RobLoxBioC ver. 0.9. R-codes are provided in Additional file 10.

### Simulated Data

The two-group simulated data were produced using the “simulateReadCounts” function in TCC [33]. The variance (*V*) of the NB distribution can generally be modeled as *V* = *μ* + *φμ*^2^. The empirical distribution of read counts to obtain the mean (*μ*) and dispersion (*φ*) parameters of the NB model was obtained from *Arabidopsis* data (three BRs for both treated and non-treated samples) in [58]. The output of the *simulateReadCounts* function is stored in the TCC class object with information about the simulated conditions and is therefore ready-to-analyze for both the DE analysis and HSC. These data were used to obtain Fig. 2, Fig. 3, and Additional file 2.

### Four RNA-Seq Data

Blekhman’s mammalian data were obtained from the supplementary website (http://genome.cshlp.org/content/suppl/2009/12/16/gr.099226.109.DC1/suppTable1.xls) [32]. The raw count matrix consisting of 20,689 genes × 36 samples (= 3 species × 2 sexes × 3 BRs × 2 technical replicates) was collapsed by summing the data for technical replicates, giving a reduced number of columns in the matrix (i.e., 18 samples; 3 species × 2 sexes × 3 BRs). These data were used to obtain Fig. 1, Fig. 3, Table 1, and Additional file 1.

Schurch’s yeast data were obtained from the GitHub website (https://github.com/bartongroup/profDGE48/tree/master/Preprocessed_data) [43]. After merging the count vectors for a total of 96 samples, data from 10 outlying samples (WT_rep21, WT_rep22, WT_rep25, WT_rep28, WT_rep34, WT_rep36, Snf2_rep06, Snf2_rep13, Snf2_rep25, and Snf2_rep35) were eliminated. Subsequent data eliminations (named no_feature, ambiguous, too_low_aQual, not_aligned, and alignment_not_unique) yielded a count matrix consisting of 7126 genes × 86 samples. These data were used to obtain Fig. 3 and Additional file 3.

Bottomly’s mouse data were [46] obtained from the ReCount website (http://bowtie-bio.sourceforge.net/recount/countTables/bottomly_count_table.txt) [45] and consisted of 36,536 genes × 21 samples. These data were used to obtain Additional file 4.

Cheung’s human data [47] were obtained from the ReCount website (http://bowtie-bio.sourceforge.net/recount/countTables/cheung_count_table.txt) [45] and consisted of 52,580 genes × 41 samples. These data were used to obtain Additional file 5.

### Two Rat Microarray Data

Nakai’s probe-level data (.CEL files) were obtained from the ArrayExpress website [59] through an R package ArrayExpress [60] by applying “GSE7623.” The MAS-quantified data were obtained using the *mas5* function in the R/Bioconductor package affy [61]. Expression signals less than 1 were set to 1 and were subsequently log_2_-transformed. The RMA-quantified data were obtained using the *rma* function in the same package, i.e., affy. The output of the function was already log_2_-transformed. The RobLoxBioC-quantified data were obtained using the *robloxbioc* function in the R package RobLoxBioC [50]. The expression signals less than 1 were set to 1 and were subsequently log_2_-transformed. These data were used to obtain Additional files 6 and 8.

Kamei’s probe-level data (.CEL files) were obtained from the ArrayExpress website [59] using the R package ArrayExpress [60] by applying “GSE30533.” The subsequent procedures were the same as those described for the Nakai data. These data were used to obtain Additional files 7 and 8.

Note that the quantification procedure was performed using R ver. 3.1.3 (affy ver. 1.44.0) because we encountered an error when executing the functions *mas5* and *robloxbioc* in R ver. 3.3.2 (affy ver. 1.52.0).

### HSC and DE Analyses

The HSC was performed using the *clusterSample* function with default options (“1 – Spearman’s *r*” as the distance and *unique* expression patterns as an objective low-count filtering method) in TCC [33]. The DE analysis was performed using three functions (calcNormFactors, estimateDE, and getResult) with default options which use functions in the package edgeR [39]. The genes were ranked in ascending order according to *p*-values. The ranks were used to calculate AUC values when analyzing simulated data (Fig. 2 and Additional file 2). The AUC values were calculated using the *AUC* function in the package ROC. The *p*-values were adjusted for multiple-testing with the Benjamini–Hochberg procedure. The adjusted *p*-values (i.e., *q*-values) were used to obtain the numbers of DEGs satisfying an arbitrarily defined FDR threshold (mainly 10%).

### Calculation of Average Silhouette (AS) Values

The AS values were calculated using the *silhouette* function in the package cluster. Examples of the procedure to estimate AS values are given in Additional file 9.

## Additional files

Additional file 1: Detailed results for Blekhman’s RNA-seq count data. (a) Silhouette indices (*s*_*i*_) for each sample *i* and the average (AS). The sample names (A1, A2, A3, B1, B2, or B3) for *i* correspond to those shown in Fig. 1b. (b) *P*_DEG_ values at various FDR thresholds (1%, 5%, 10%, 20%, 30%, and 40% FDR). The values at 10% FDR were the same as those shown in Fig. 1b. (c) Percentages of true DEGs (*P*_trueDEG_), defined as *P*_DEG_ × (1 − FDR threshold), at corresponding FDR thresholds shown in (b). (XLSX 19 kb)

Additional file 2: Effects of *N*_rep_ on parameter estimates (simulated count data). Bootstrapping results for simulated data under different *P*_simDEG_ values are shown: *P*_simDEG_ = 10% (Page 1), 5% (Page 2), 2% (Page 3), 1% (Page 4), 0.5% (Page 5), 0.1% (Page 6), and 0.02% (Page 7). Other legends are the same as those in Fig. 2. (PPTX 110 kb)

Additional file 3: Results for Schurch’s RNA-seq count data. For (a–b), Bootstrapping results for Schurch data comparing 42 wild-type samples and 44 Δsnf2 mutant samples are shown. Legends are the same as those in Fig. 2. (c) HSC dendrogram. Two distinct clusters, a wild-type cluster (right side) and Δsnf2 mutant cluster (left side), can be seen. The intra-group distances within 42 wild-type samples and 44 Δsnf2 mutant samples were 0.0144 and 0.0084, respectively. (d) Scatter plots of *P*_DEG_ vs. AS at *N*_rep_ = 3 (black), 6 (blue), and 9 (sky blue). (PPTX 65 kb)

Additional file 4: Results for Bottomly’s RNA-seq count data. For (a–b), Bootstrapping results for Bottomly data comparing 10 C57BL/6J strains (A1, A2 …, A10) vs. 11 DBA/2 J strains (B1, B2, …, B11) are shown. (c) HSC dendrogram. For explanation, four clusters are defined in (d) the HSC dendrogram: the *B1* cluster (consisting of B1, B2, B3, and B8), *A8* cluster (A8, A9, and A10), *A2* cluster (A2, A4, and A6), and *B4* cluster (B4, B5, B6, B7, B9, B10, and B11). (d) Scatter plots of *P*_DEG_ vs. AS at *N*_rep_ = 3 (black), 6 (blue), and 9 (sky blue). (PPTX 55 kb)

Additional file 5: Results for Cheung’s RNA-seq count data. For (a–b), Bootstrapping results for Cheung data comparing 17 females (A1, A2, …, A17) vs. 24 males (B1, B2, …, B24) are shown. (c) HSC dendrogram. (d) Scatter plots of *P*_DEG_ vs. AS at *N*_rep_ = 3 (black), 6 (blue), and 9 (sky blue). (PPTX 58 kb)

Additional file 6: Results for Nakai’s microarray data. (a) HSC dendrogram for Nakai data consisting of 31,099 *genes* × 24 samples and (b) *P*_DEG_ and AS values from a total of 15 two-group comparisons with *N*_rep_ = 4 are shown: MAS-quantified data (Page 1), RMA-quantified data (Page 2), and RobLoxBioC-quantified data (Page 3). (PPTX 76 kb)

Additional file 7: Results for Kamei’s microarray data. HSC dendrograms for (a) MAS-, (b) RMA-, and (c) RobLoxBioC-quantified data are shown. These data consist of 31,099 *genes* × 10 samples and compares two conditions (five *Iron_def* samples vs. five *Control* samples). The *P*_DEG_ and AS values are also shown on the right side of the dendrogram. (PPTX 49 kb)

Additional file 8: HSC dendrograms for merged microarray data (Nakai + Kamei). HSC dendrograms for (a) MAS-, (b) RMA-, and (c) RobLoxBioC-quantified data are shown. These data consist of 31,099 *genes* × 34 samples (24 from Nakai and 10 from Kamei data). (PPTX 62 kb)

Additional file 9: Examples of AS estimates for two- and three-group data. The procedures for analyzing Nakai’s MAS-quantified data consisting of 31,099 probesets × 24 samples are provided. Example 1 compares three-group data with four BRs, 4 *BAT_fed* samples vs. 4 *WAT_fed* samples vs. 4 *LIV_fed* samples, with AS = 0.460. Example 2 compares three-group data with two BRs, “*BAT_fed1* and *2*” vs. “*WAT_fed1* and *2*” vs. “*LIV_fed1* and *2*,” with AS = 0.438. Example 3 compares three-group data with two BRs, “*BAT_fed1* and *BAT_fas1*” vs. “*BAT_fed2* and *BAT_fas2*” vs. “*BAT_fed3* and *BAT_fas3*,” with AS = − 0.185. Example 4 compares two-group data with four BRs, 4 *BAT_fed* samples vs. 4 *WAT_fed* samples, with AS = 0.374. Example 5 compares two-group data with four BRs, 4 *BAT_fed* samples vs. 4 *LIV_fed* samples, with AS = 0.657. (R 3 kb)

Additional file 10: R-codes for analyses. This zipped file includes a total of 23 R-code files. Results can be obtained by executing scripts in the order of the serial numbers *XX* in the filename “rcode_*XX*_...” Note that two files (“rcode_08_Add6_pre.R” and “rcode_10_Add7_pre.R”) must be executed using R ver. 3.1.3 (affy ver. 1.44.0) instead of R ver. 3.3.2 (affy ver. 1.52.0). (ZIP 33 kb)

## Abbreviations

AS: Average silhouette
AUC: the area under the ROC curve
BAT: Brown adipose tissue
BR: Biological replicate
BV: Biological variation
DE: Differential expression
DEG: Differentially expressed gene
F: Female
FDR: False discovery rate
*H*_*0*_: null hypothesis
HS: *Homo sapiens*
HSC: Hierarchical sample clustering
LIV: Liver tissue
M: Male
NB: Negative binomial (distribution or model)
*N*_rep_: Number of (biological) replicates
*P*_DEG_: Percentage of estimated DEGs (satisfying basically 10% FDR) by TCC
*P*_simDEG_: Percentage of DEGs when generating simulated data
*P*_trueDEG_: Percentage of true DEGs defined as *P*_DEG_ × (1.0 – FDR threshold)
PT: *Pan troglodytes*
RM: *Rhesus macaques*
ROC: Receiver operating characteristic
SC: Sample clustering
TCC: Tag Count Comparison
*V*: Variance
WAT: White adipose tissue
