# Supplementary material for: Silhouette Scores for Arbitrary Defined Groups in Gene Expression Data and Insights into Differential Expression Results
Source: Biol Proced Online. 2018 Mar 1;20:5. doi: 10.1186/s12575-018-0067-8 (PMC5831220; doi:10.1186/s12575-018-0067-8)
Supplement: Supplementary file 2 — Effects of Nrep on parameter estimates (simulated count data). Bootstrapping results for simulated data under different PsimDEG values are shown: PsimDEG = 10% (Page 1), 5% (Page 2), 2% (Page 3), 1% (Page 4), 0.5% (Page 5), 0.1% (Page 6), and 0.02% (Page 7). Other legends are the same as those in Fig. 2. (PPTX 110 kb) [file 12575_2018_67_MOESM2_ESM.pptx]

## Slide 1
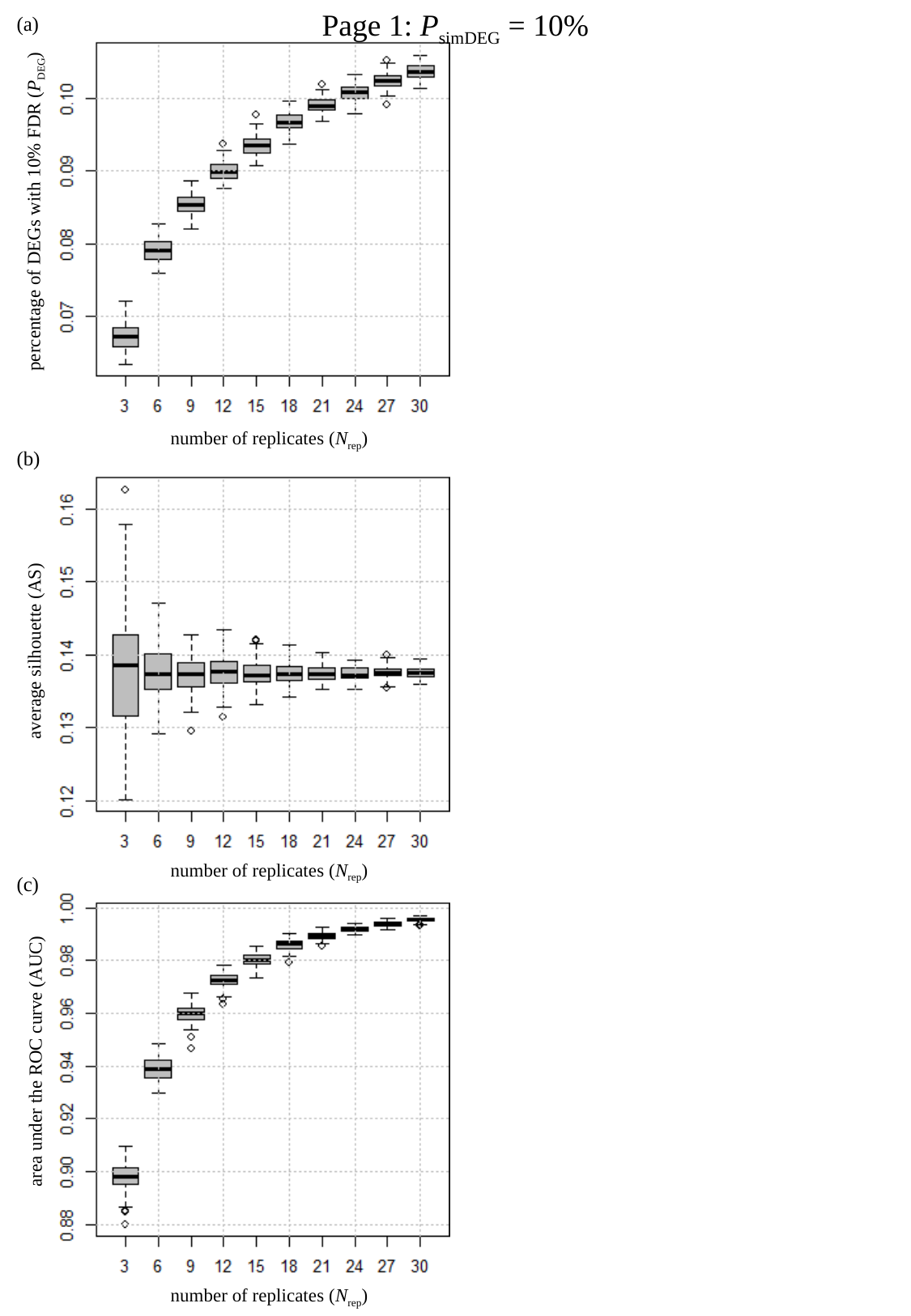

Page 1: PsimDEG = 10%
(a)
percentage of DEGs with 10% FDR (PDEG)
number of replicates (Nrep)
(b)
average silhouette (AS)
number of replicates (Nrep)
(c)
area under the ROC curve (AUC)
number of replicates (Nrep)

## Slide 2
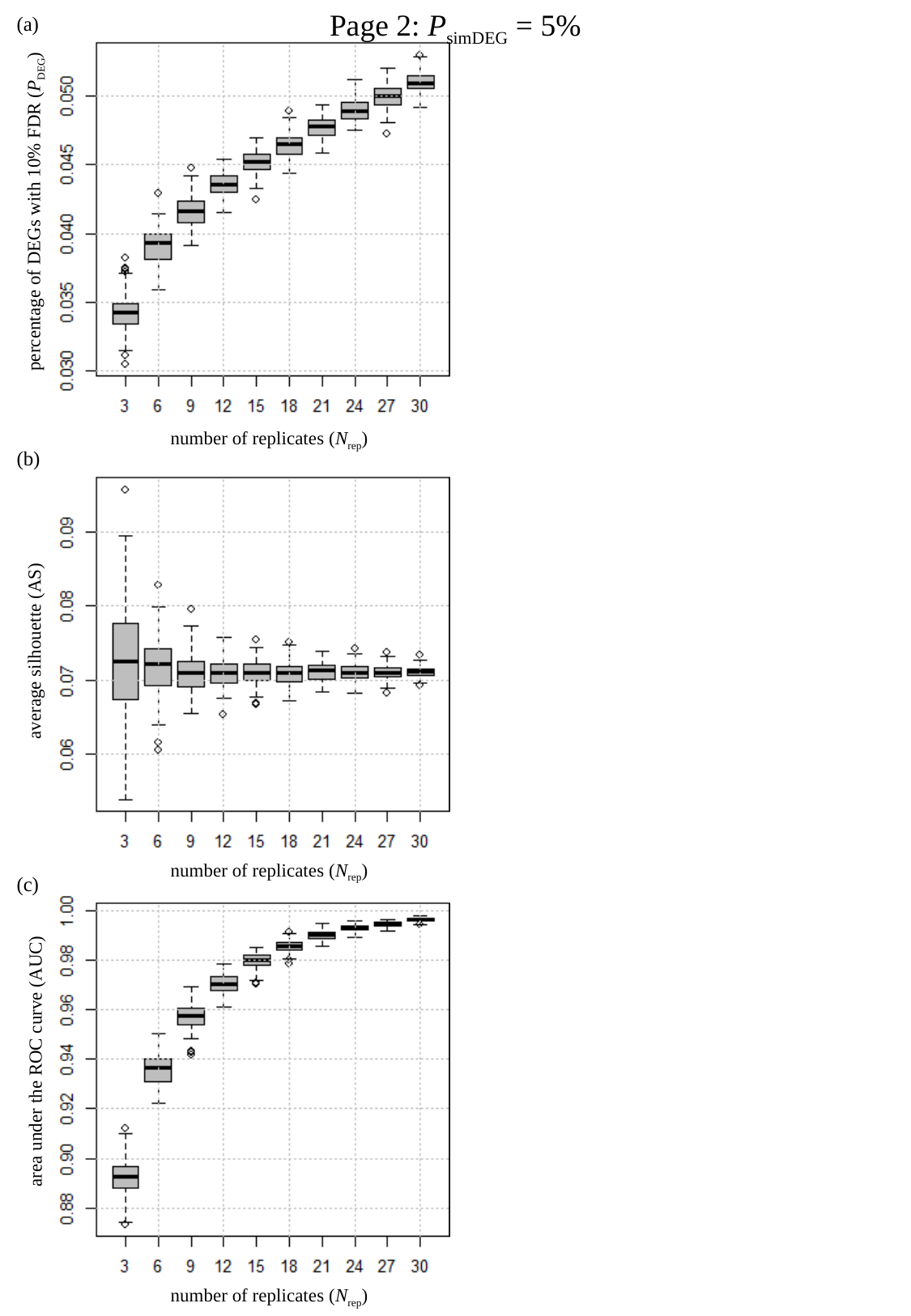

Page 2: PsimDEG = 5%
(a)
percentage of DEGs with 10% FDR (PDEG)
number of replicates (Nrep)
(b)
average silhouette (AS)
number of replicates (Nrep)
(c)
area under the ROC curve (AUC)
number of replicates (Nrep)

## Slide 3
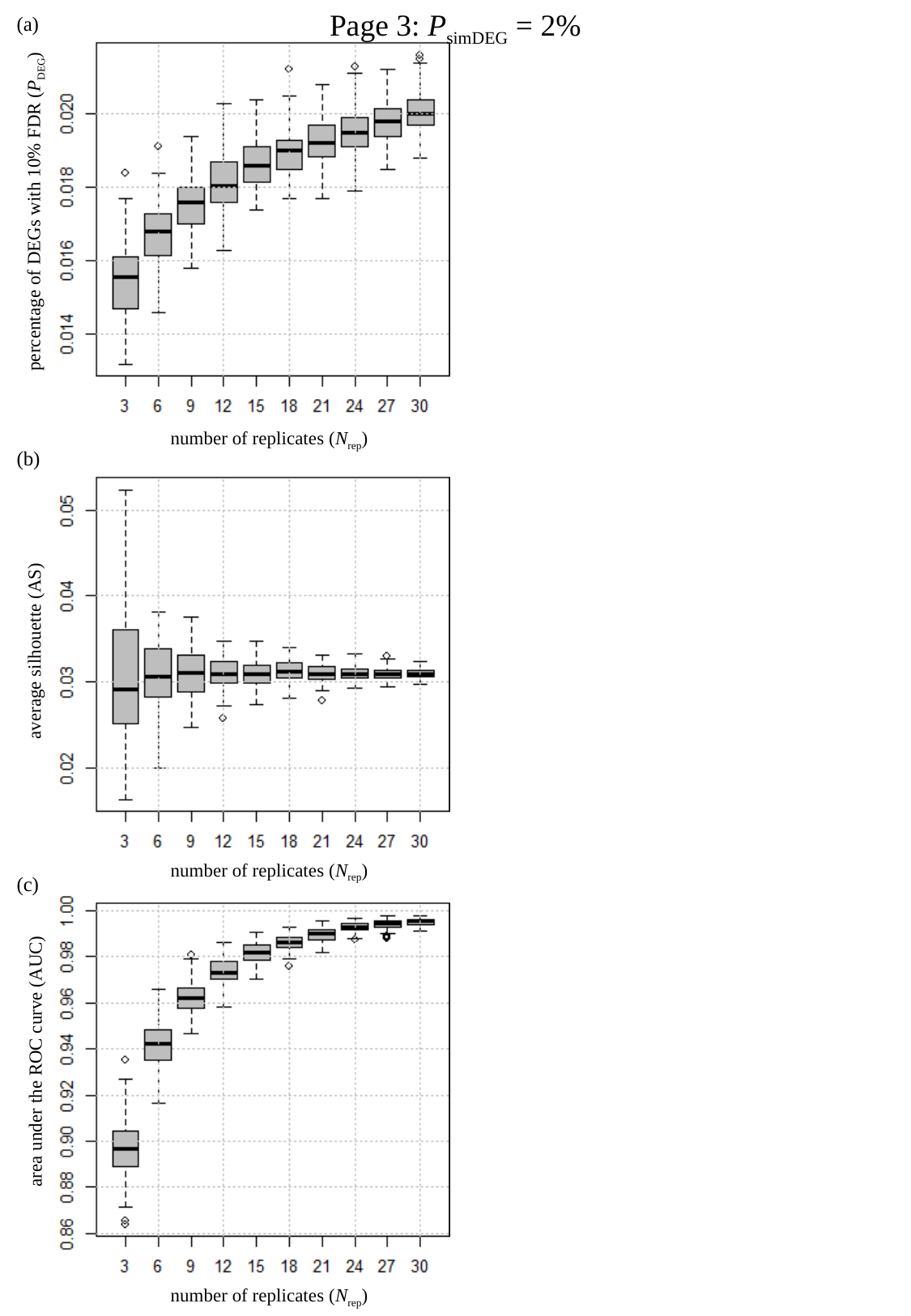

Page 3: PsimDEG = 2%
(a)
percentage of DEGs with 10% FDR (PDEG)
number of replicates (Nrep)
(b)
average silhouette (AS)
number of replicates (Nrep)
(c)
area under the ROC curve (AUC)
number of replicates (Nrep)

## Slide 4
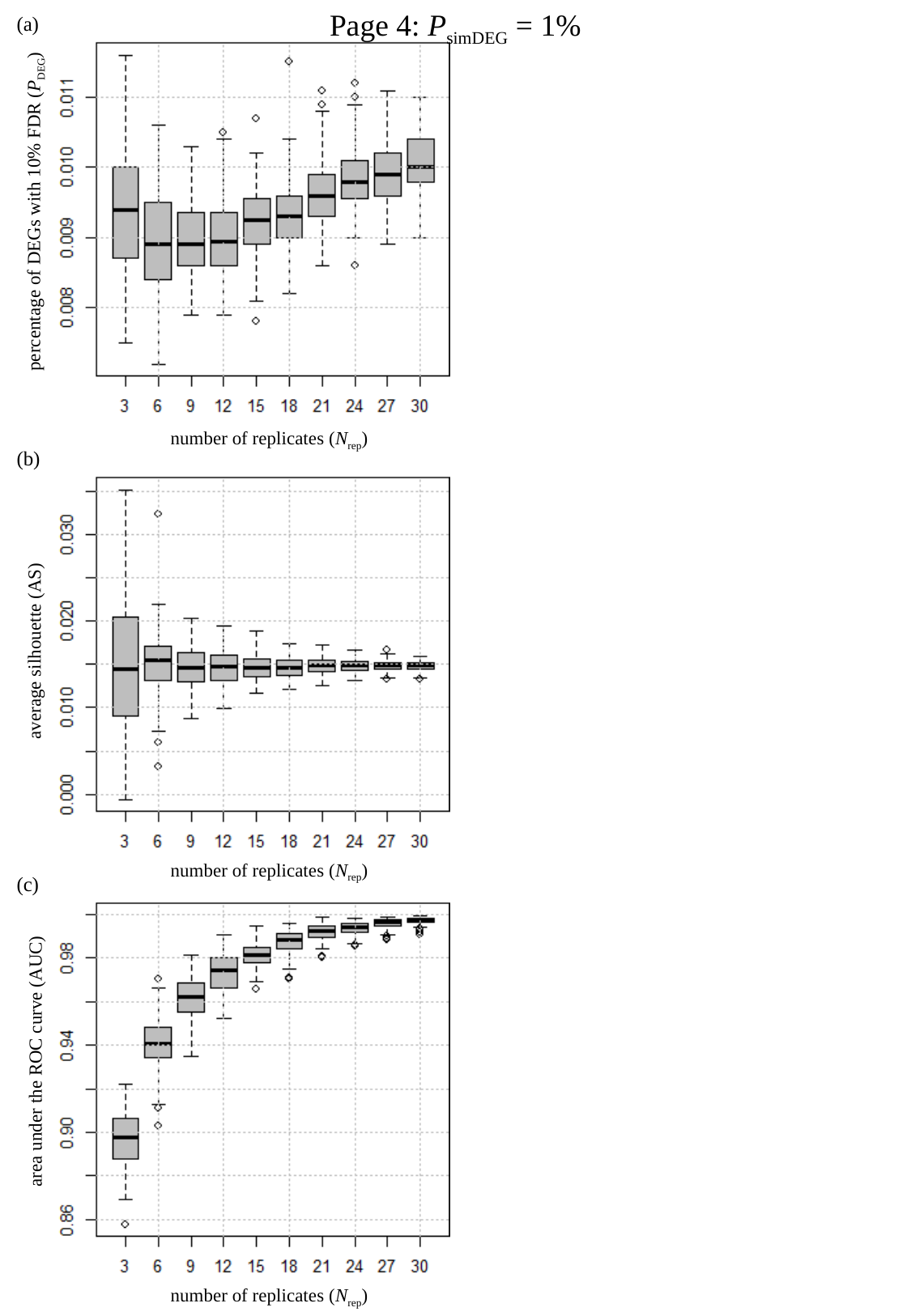

Page 4: PsimDEG = 1%
(a)
percentage of DEGs with 10% FDR (PDEG)
number of replicates (Nrep)
(b)
average silhouette (AS)
number of replicates (Nrep)
(c)
area under the ROC curve (AUC)
number of replicates (Nrep)

## Slide 5
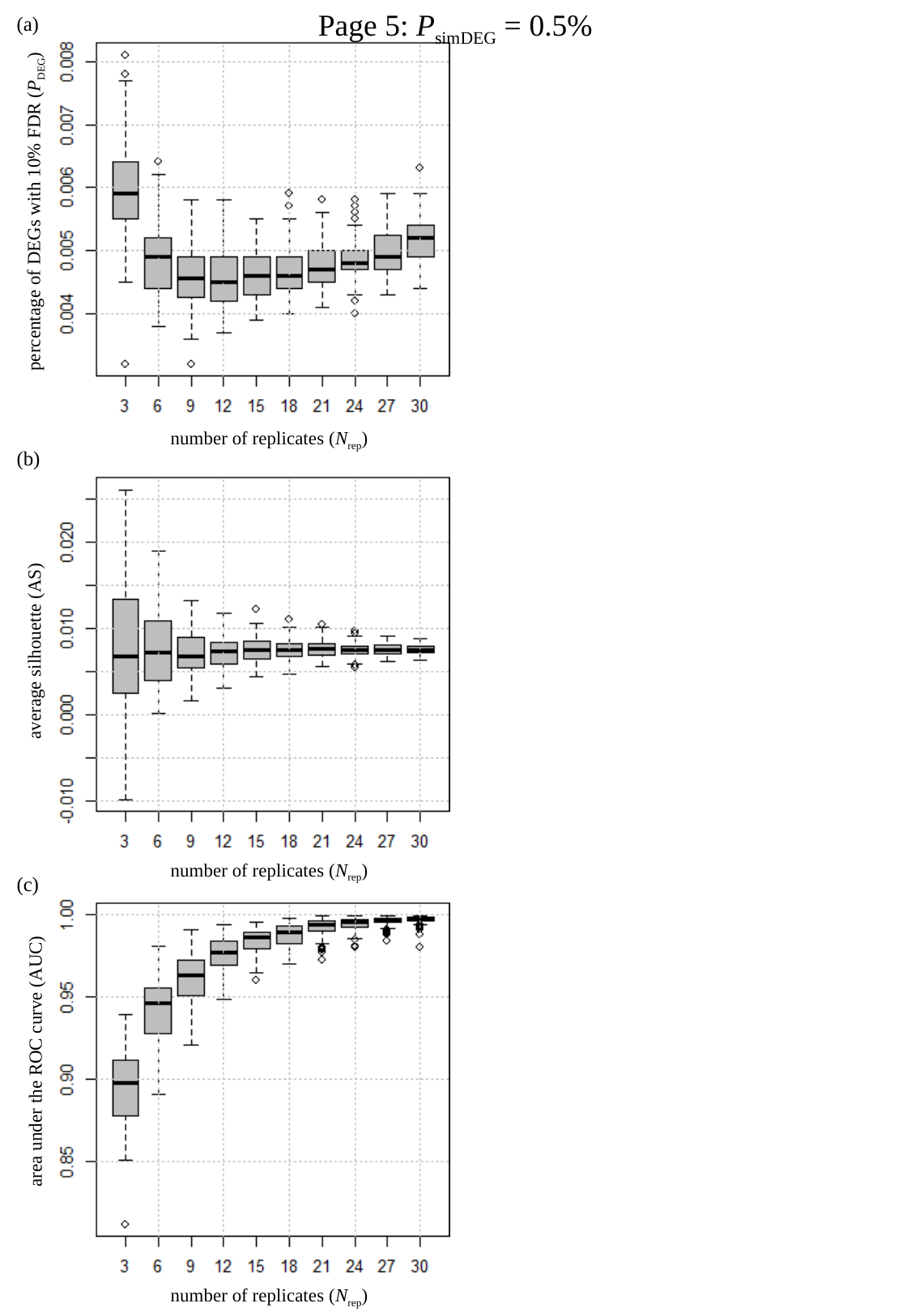

Page 5: PsimDEG = 0.5%
(a)
percentage of DEGs with 10% FDR (PDEG)
number of replicates (Nrep)
(b)
average silhouette (AS)
number of replicates (Nrep)
(c)
area under the ROC curve (AUC)
number of replicates (Nrep)

## Slide 6
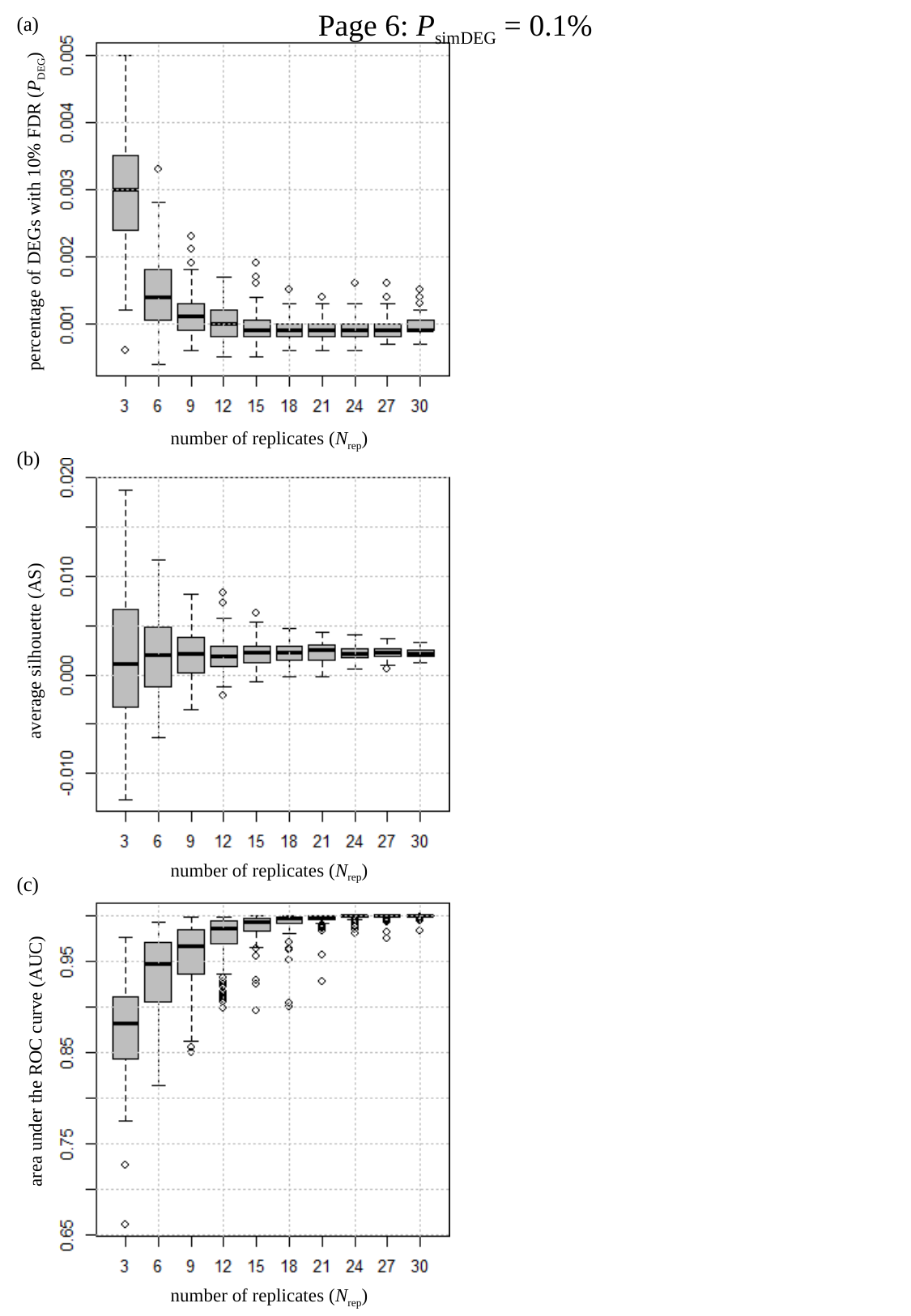

Page 6: PsimDEG = 0.1%
(a)
percentage of DEGs with 10% FDR (PDEG)
number of replicates (Nrep)
(b)
average silhouette (AS)
number of replicates (Nrep)
(c)
area under the ROC curve (AUC)
number of replicates (Nrep)

## Slide 7
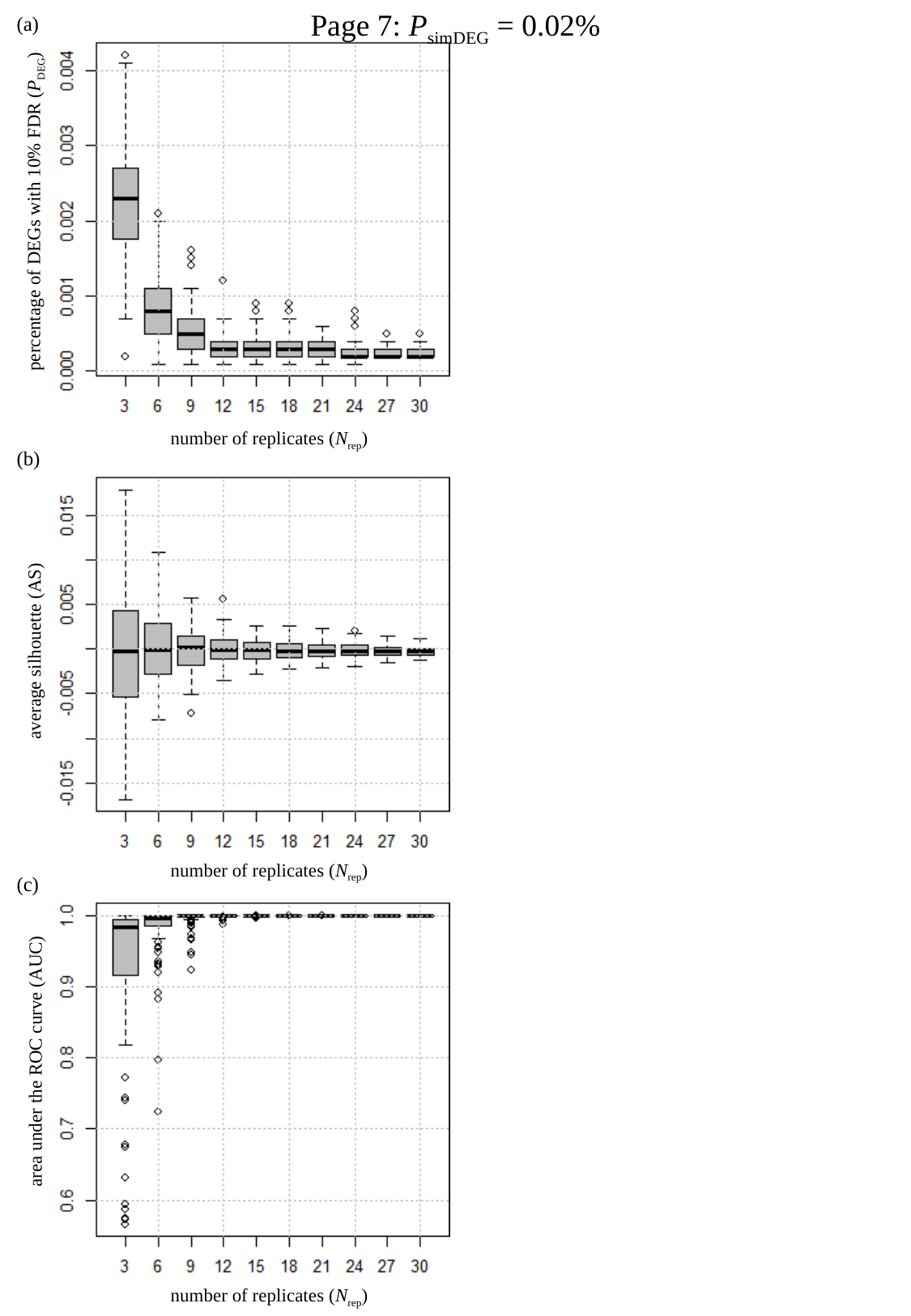

Page 7: PsimDEG = 0.02%
(a)
percentage of DEGs with 10% FDR (PDEG)
number of replicates (Nrep)
(b)
average silhouette (AS)
number of replicates (Nrep)
(c)
area under the ROC curve (AUC)
number of replicates (Nrep)
