# Supplementary material for: Silhouette Scores for Arbitrary Defined Groups in Gene Expression Data and Insights into Differential Expression Results
Source: Biol Proced Online. 2018 Mar 1;20:5. doi: 10.1186/s12575-018-0067-8 (PMC5831220; doi:10.1186/s12575-018-0067-8)
Supplement: Supplementary file 3 — Results for Schurch’s RNA-seq count data. For (a–b), Bootstrapping results for Schurch data comparing 42 wild-type samples and 44 Δsnf2 mutant samples are shown. Legends are the same as those in Fig. 2. (c) HSC dendrogram. Two distinct clusters, a wild-type cluster (right side) and Δsnf2 mutant cluster (left side), can be seen. The intra-group distances within 42 wild-type samples and 44 Δsnf2 mutant samples were 0.0144 and 0.0084, respectively. (d) Scatter plots of PDEG vs. AS at Nrep = 3 (black), 6 (blue), and 9 (sky blue). (PPTX 65 kb) [file 12575_2018_67_MOESM3_ESM.pptx]

## Slide 1
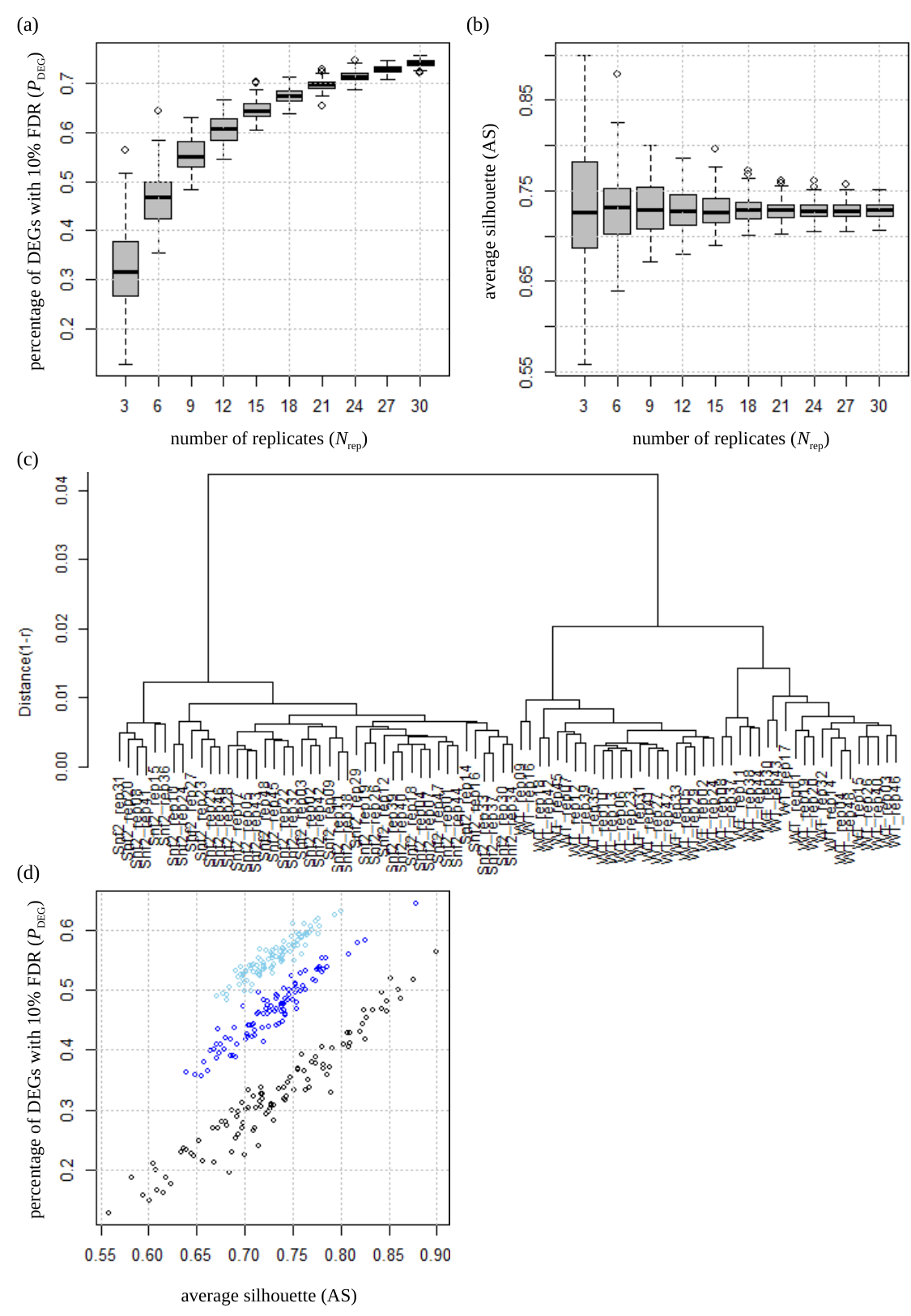

(a)
(b)
percentage of DEGs with 10% FDR (PDEG)
average silhouette (AS)
number of replicates (Nrep)
number of replicates (Nrep)
(c)
(d)
percentage of DEGs with 10% FDR (PDEG)
average silhouette (AS)
