# Supplementary material for: Silhouette Scores for Arbitrary Defined Groups in Gene Expression Data and Insights into Differential Expression Results
Source: Biol Proced Online. 2018 Mar 1;20:5. doi: 10.1186/s12575-018-0067-8 (PMC5831220; doi:10.1186/s12575-018-0067-8)
Supplement: Supplementary file 4 — Results for Bottomly’s RNA-seq count data. For (a–b), Bootstrapping results for Bottomly data comparing 10 C57BL/6J strains (A1, A2 …, A10) vs. 11 DBA/2 J strains (B1, B2, …, B11) are shown. (c) HSC dendrogram. For explanation, four clusters are defined in (d) the HSC dendrogram: the B1 cluster (consisting of B1, B2, B3, and B8), A8 cluster (A8, A9, and A10), A2 cluster (A2, A4, and A6), and B4 cluster (B4, B5, B6, B7, B9, B10, and B11). (d) Scatter plots of PDEG vs. AS at Nrep = 3 (black), 6 (blue), and 9 (sky blue). (PPTX 55 kb) [file 12575_2018_67_MOESM4_ESM.pptx]

## Slide 1
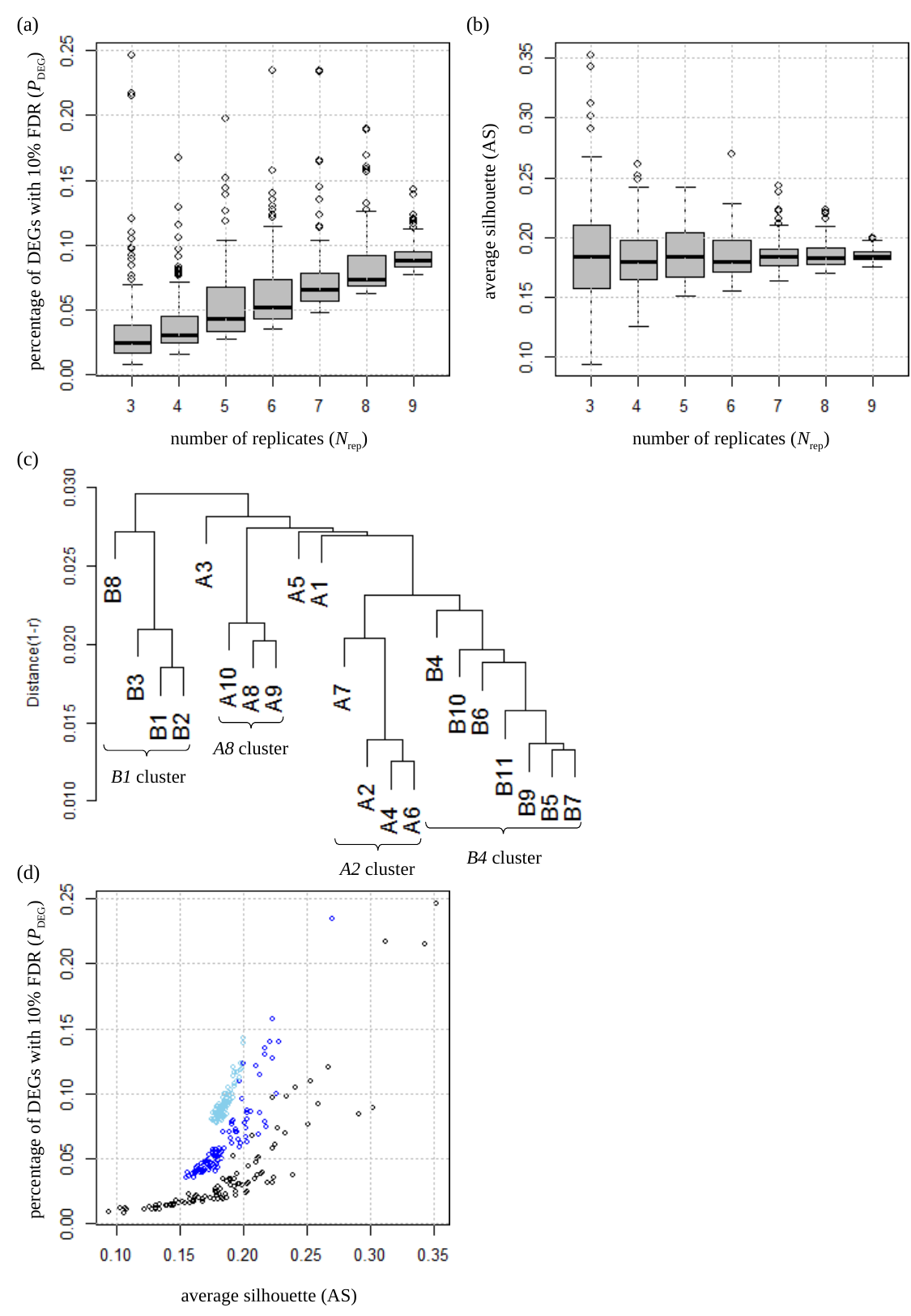

(a)
(b)
percentage of DEGs with 10% FDR (PDEG)
average silhouette (AS)
number of replicates (Nrep)
number of replicates (Nrep)
(c)
A8 cluster
B1 cluster
B4 cluster
(d)
A2 cluster
percentage of DEGs with 10% FDR (PDEG)
average silhouette (AS)
