# Supplementary material for: Silhouette Scores for Arbitrary Defined Groups in Gene Expression Data and Insights into Differential Expression Results
Source: Biol Proced Online. 2018 Mar 1;20:5. doi: 10.1186/s12575-018-0067-8 (PMC5831220; doi:10.1186/s12575-018-0067-8)
Supplement: Supplementary file 5 — Results for Cheung’s RNA-seq count data. For (a–b), Bootstrapping results for Cheung data comparing 17 females (A1, A2, …, A17) vs. 24 males (B1, B2, …, B24) are shown. (c) HSC dendrogram. (d) Scatter plots of PDEG vs. AS at Nrep = 3 (black), 6 (blue), and 9 (sky blue). (PPTX 58 kb) [file 12575_2018_67_MOESM5_ESM.pptx]

## Slide 1
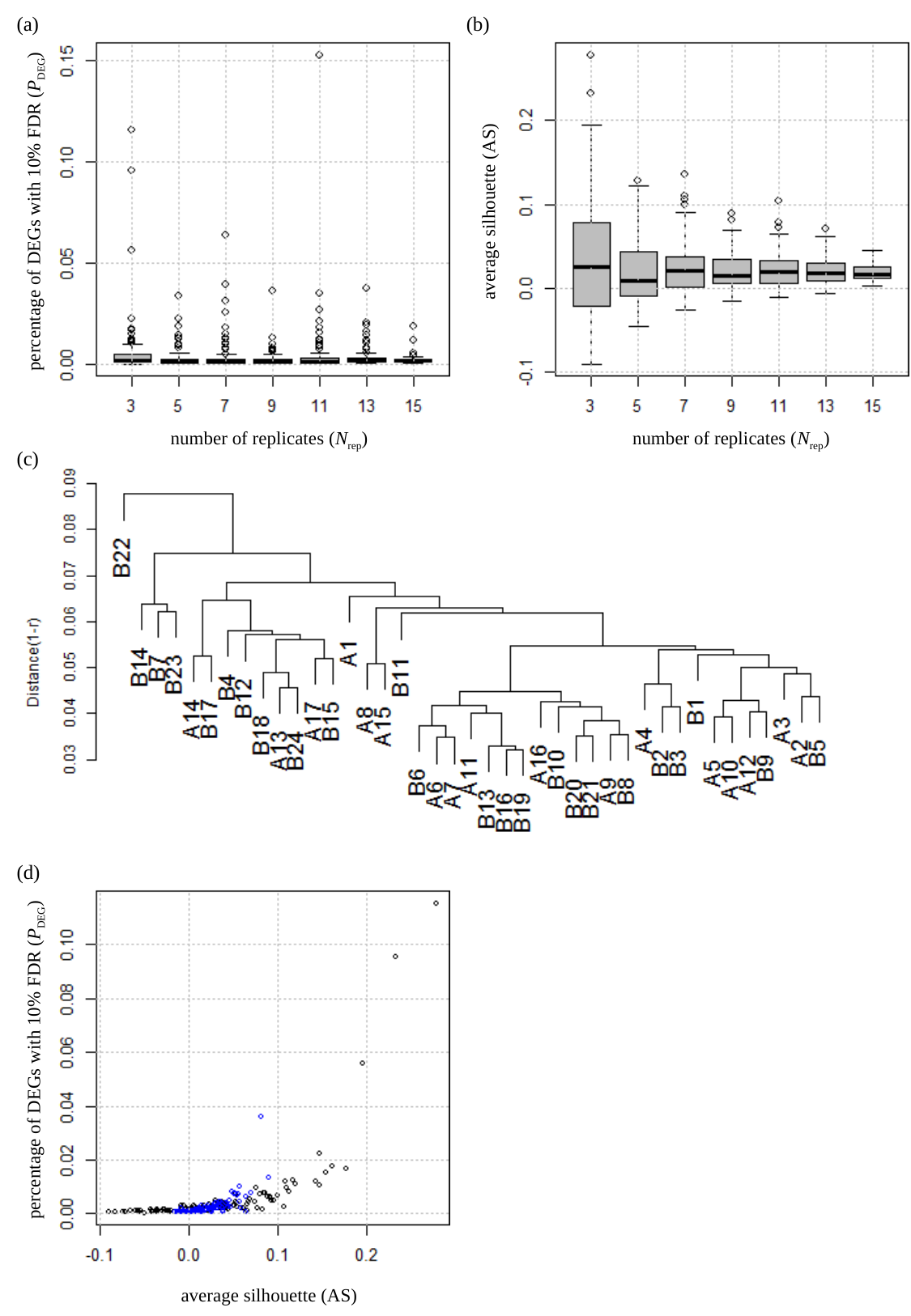

(a)
(b)
percentage of DEGs with 10% FDR (PDEG)
average silhouette (AS)
number of replicates (Nrep)
number of replicates (Nrep)
(c)
(d)
percentage of DEGs with 10% FDR (PDEG)
average silhouette (AS)

## Slide 2
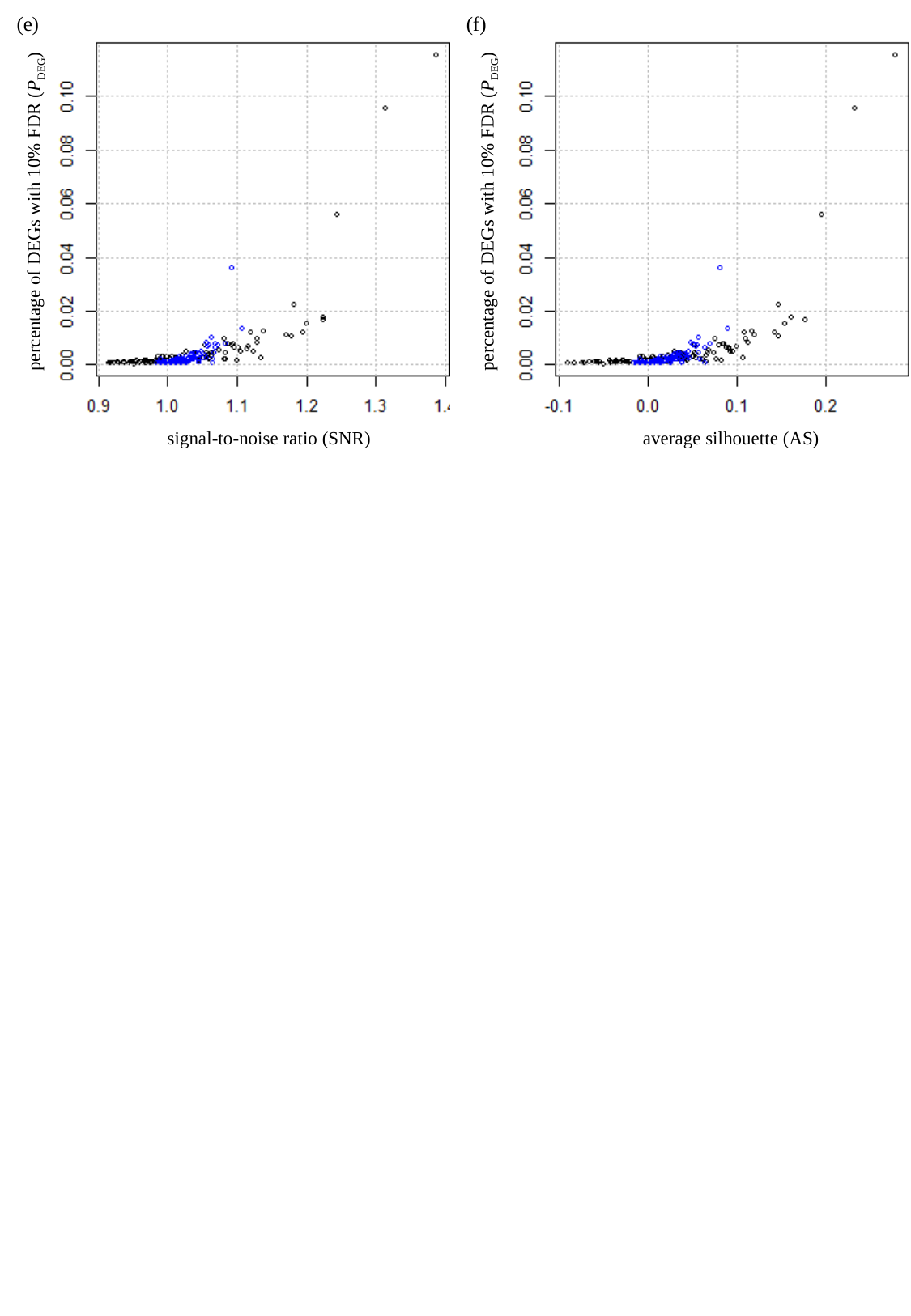

(e)
(f)
percentage of DEGs with 10% FDR (PDEG)
percentage of DEGs with 10% FDR (PDEG)
signal-to-noise ratio (SNR)
average silhouette (AS)
