# Supplementary material for: Silhouette Scores for Arbitrary Defined Groups in Gene Expression Data and Insights into Differential Expression Results
Source: Biol Proced Online. 2018 Mar 1;20:5. doi: 10.1186/s12575-018-0067-8 (PMC5831220; doi:10.1186/s12575-018-0067-8)
Supplement: Supplementary file 6 — Results for Nakai’s microarray data. (a) HSC dendrogram for Nakai data consisting of 31,099 genes × 24 samples and (b) PDEG and AS values from a total of 15 two-group comparisons with Nrep = 4 are shown: MAS-quantified data (Page 1), RMA-quantified data (Page 2), and RobLoxBioC-quantified data (Page 3). (PPTX 76 kb) [file 12575_2018_67_MOESM6_ESM.pptx]

## Slide 1
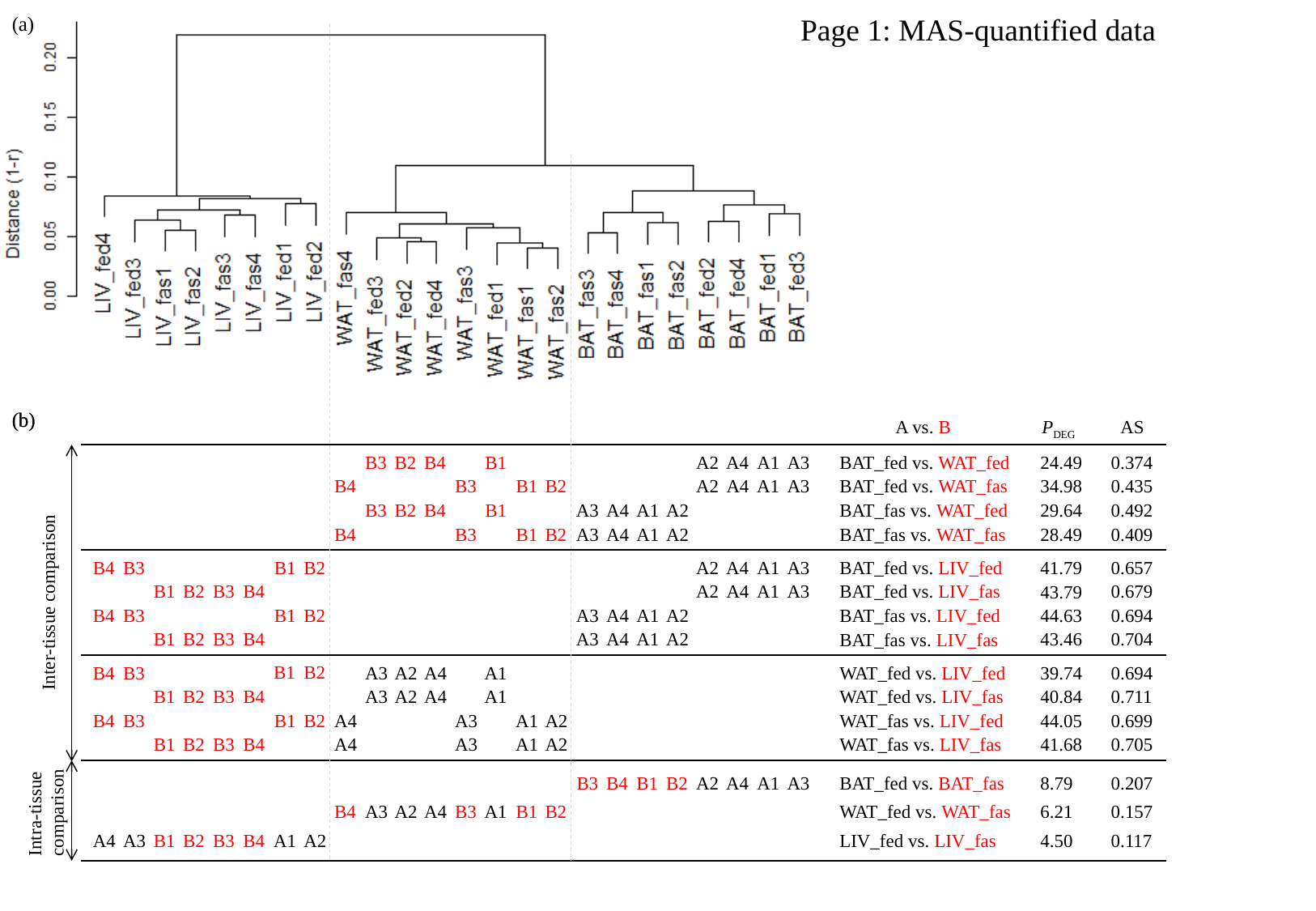

(a)
Page 1: MAS-quantified data
(b)
(b)
A vs. B
PDEG
AS
B1
B3
B2
B4
A2
A4
A1
A3
BAT_fed vs. WAT_fed
24.49
0.374
B4
B3
B1
B2
A2
A4
A1
A3
BAT_fed vs. WAT_fas
34.98
0.435
B3
B2
B4
B1
A3
A4
A1
A2
BAT_fas vs. WAT_fed
29.64
0.492
B4
B3
B1
B2
A3
A4
A1
A2
BAT_fas vs. WAT_fas
28.49
0.409
A2
A4
A1
A3
B1
B2
BAT_fed vs. LIV_fed
41.79
0.657
B4
B3
A2
A4
A1
A3
B1
B2
B3
B4
BAT_fed vs. LIV_fas
0.679
43.79
Inter-tissue comparison
B1
B4
B3
B2
A3
A4
A1
A2
BAT_fas vs. LIV_fed
0.694
44.63
A3
A4
A1
A2
B1
B2
B3
B4
43.46
0.704
BAT_fas vs. LIV_fas
B1
B2
A3
A2
A4
A1
WAT_fed vs. LIV_fed
39.74
0.694
B4
B3
B1
B2
B3
B4
WAT_fed vs. LIV_fas
0.711
40.84
A3
A2
A4
A1
WAT_fas vs. LIV_fed
B1
B4
B3
B2
A4
A3
A1
A2
44.05
0.699
B1
B2
B3
B4
A4
A3
A1
A2
WAT_fas vs. LIV_fas
41.68
0.705
B3
B4
B1
B2
A2
A4
A1
A3
BAT_fed vs. BAT_fas
8.79
0.207
Intra-tissue comparison
B4
A3
A2
A4
B3
A1
B1
B2
WAT_fed vs. WAT_fas
6.21
0.157
B1
B2
B3
B4
A1
LIV_fed vs. LIV_fas
4.50
0.117
A4
A3
A2

## Slide 2
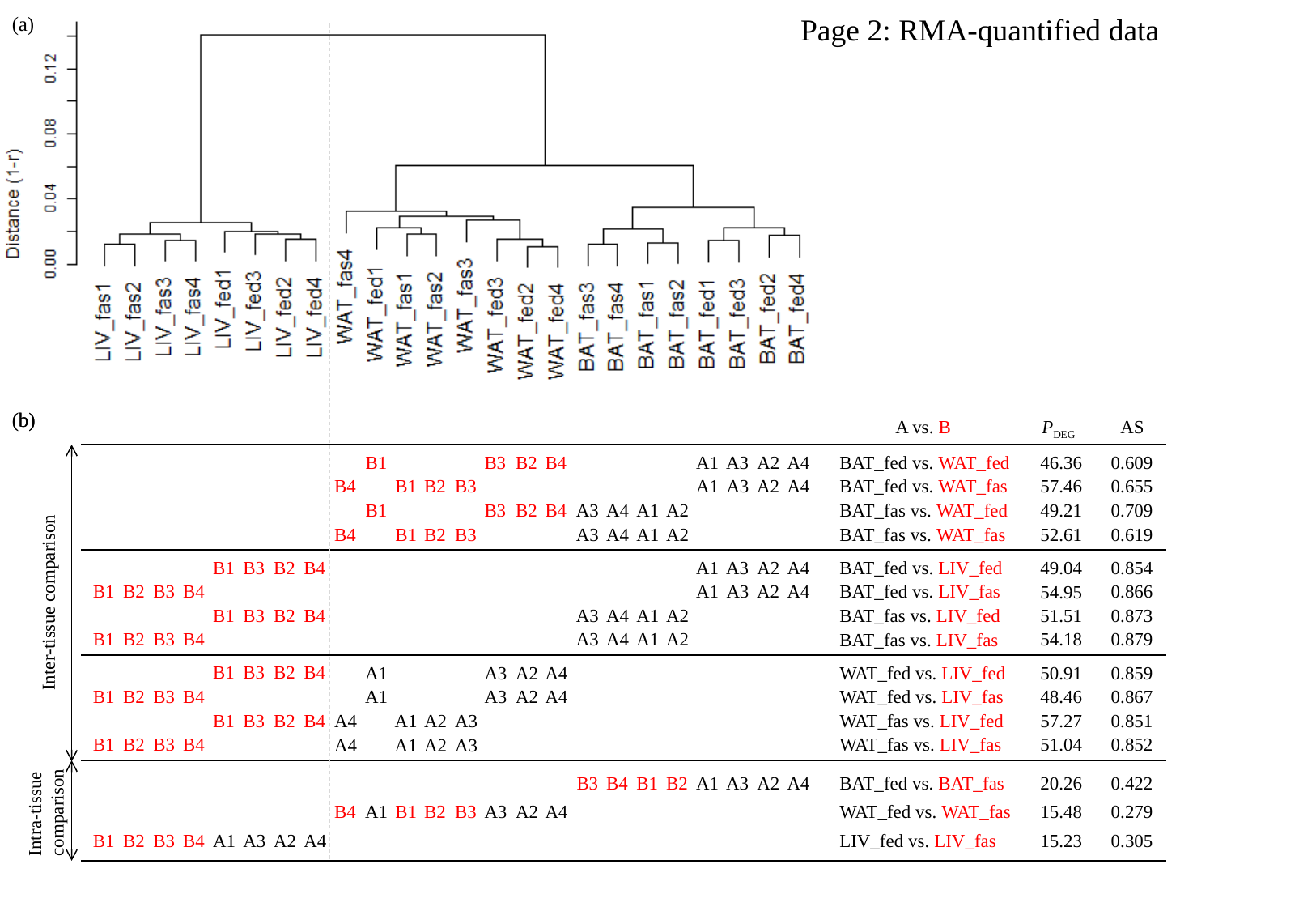

(a)
Page 2: RMA-quantified data
(b)
(b)
A vs. B
PDEG
AS
B1
B3
B2
B4
A1
A3
A2
A4
BAT_fed vs. WAT_fed
46.36
0.609
B4
B1
B2
B3
A1
A3
A2
A4
BAT_fed vs. WAT_fas
57.46
0.655
B1
B3
B2
B4
A3
A4
A1
A2
BAT_fas vs. WAT_fed
49.21
0.709
B4
B1
B2
B3
A3
A4
A1
A2
BAT_fas vs. WAT_fas
52.61
0.619
A1
A3
A2
A4
B1
B3
B2
B4
BAT_fed vs. LIV_fed
49.04
0.854
A1
A3
A2
A4
B1
B2
B3
B4
BAT_fed vs. LIV_fas
0.866
54.95
Inter-tissue comparison
B1
B3
B2
B4
A3
A4
A1
A2
BAT_fas vs. LIV_fed
0.873
51.51
A3
A4
A1
A2
B1
B2
B3
B4
54.18
0.879
BAT_fas vs. LIV_fas
B1
B3
B2
B4
A1
A3
A2
A4
WAT_fed vs. LIV_fed
50.91
0.859
B1
B2
B3
B4
A1
A3
A2
A4
WAT_fed vs. LIV_fas
0.867
48.46
WAT_fas vs. LIV_fed
B1
B3
B2
B4
A4
A1
A2
A3
57.27
0.851
B1
B2
B3
B4
WAT_fas vs. LIV_fas
51.04
0.852
A4
A1
A2
A3
B3
B4
B1
B2
A1
A3
A2
A4
BAT_fed vs. BAT_fas
20.26
0.422
Intra-tissue comparison
B4
B1
B2
B3
A1
A3
A2
A4
WAT_fed vs. WAT_fas
15.48
0.279
B1
B2
B3
B4
A1
A3
A2
A4
LIV_fed vs. LIV_fas
15.23
0.305

## Slide 3
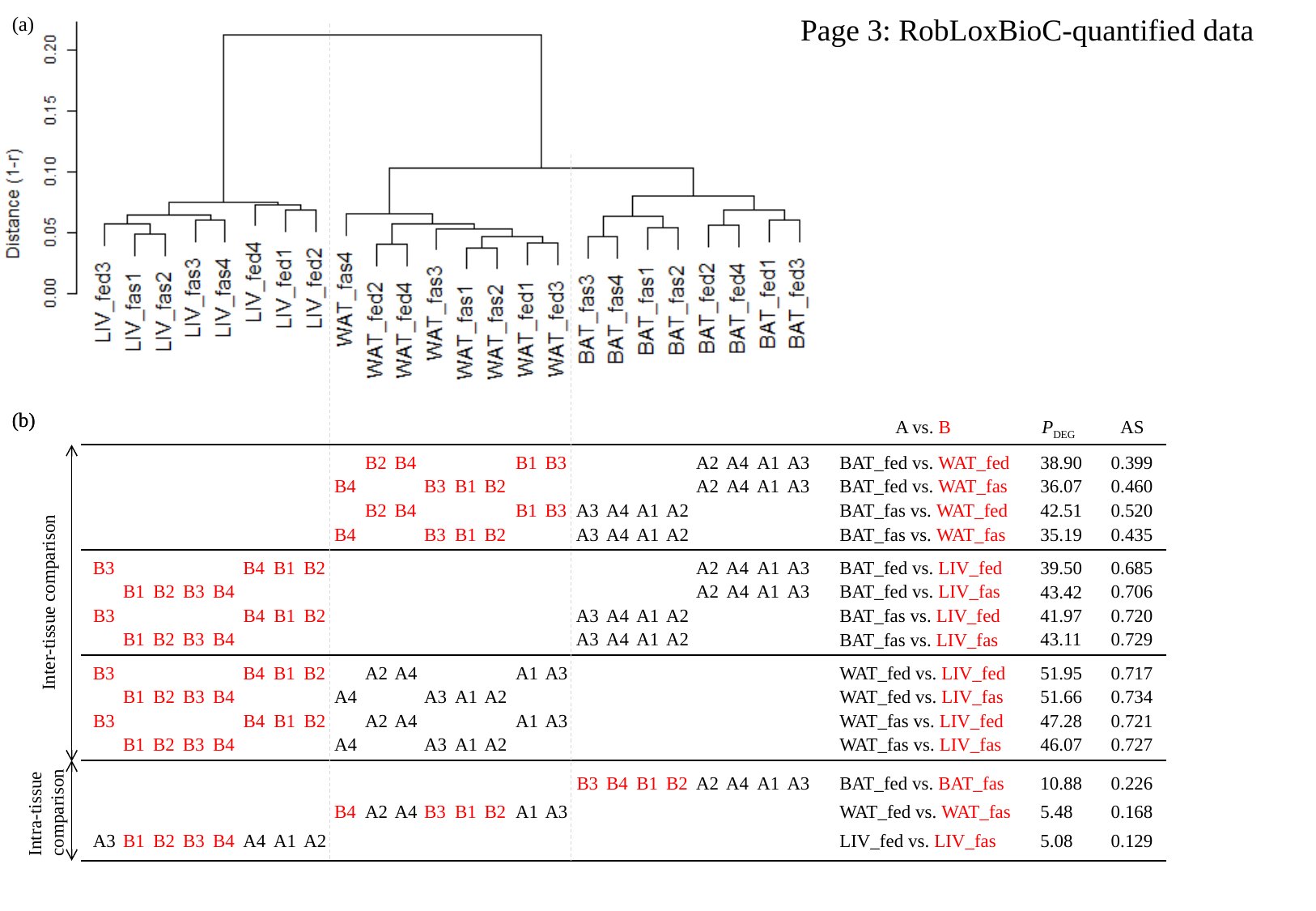

(a)
Page 3: RobLoxBioC-quantified data
(b)
(b)
A vs. B
PDEG
AS
B2
B4
B1
B3
A2
A4
A1
A3
BAT_fed vs. WAT_fed
38.90
0.399
B4
B3
B1
B2
A2
A4
A1
A3
BAT_fed vs. WAT_fas
36.07
0.460
B2
B4
B1
B3
A3
A4
A1
A2
BAT_fas vs. WAT_fed
42.51
0.520
B4
B3
B1
B2
A3
A4
A1
A2
BAT_fas vs. WAT_fas
35.19
0.435
A2
A4
A1
A3
B3
B4
B1
B2
BAT_fed vs. LIV_fed
39.50
0.685
A2
A4
A1
A3
B1
B2
B3
B4
BAT_fed vs. LIV_fas
0.706
43.42
Inter-tissue comparison
B3
B4
B1
B2
A3
A4
A1
A2
BAT_fas vs. LIV_fed
0.720
41.97
B1
B2
B3
B4
A3
A4
A1
A2
43.11
0.729
BAT_fas vs. LIV_fas
B3
B4
B1
B2
A2
A4
A1
A3
WAT_fed vs. LIV_fed
51.95
0.717
B1
B2
B3
B4
A4
A3
A1
A2
WAT_fed vs. LIV_fas
0.734
51.66
B3
B4
B1
B2
WAT_fas vs. LIV_fed
A2
A4
A1
A3
47.28
0.721
B1
B2
B3
B4
A4
A3
A1
A2
WAT_fas vs. LIV_fas
46.07
0.727
B3
B4
B1
B2
A2
A4
A1
A3
BAT_fed vs. BAT_fas
10.88
0.226
Intra-tissue comparison
B4
B3
B1
B2
A2
A4
A1
A3
WAT_fed vs. WAT_fas
5.48
0.168
A3
B1
B2
B3
B4
A4
A1
A2
LIV_fed vs. LIV_fas
5.08
0.129
