# Supplementary material for: Silhouette Scores for Arbitrary Defined Groups in Gene Expression Data and Insights into Differential Expression Results
Source: Biol Proced Online. 2018 Mar 1;20:5. doi: 10.1186/s12575-018-0067-8 (PMC5831220; doi:10.1186/s12575-018-0067-8)
Supplement: Supplementary file 7 — Results for Kamei’s microarray data. HSC dendrograms for (a) MAS-, (b) RMA-, and (c) RobLoxBioC-quantified data are shown. These data consist of 31,099 genes × 10 samples and compares two conditions (five Iron_def samples vs. five Control samples). The PDEG and AS values are also shown on the right side of the dendrogram. (PPTX 49 kb) [file 12575_2018_67_MOESM7_ESM.pptx]

## Slide 1
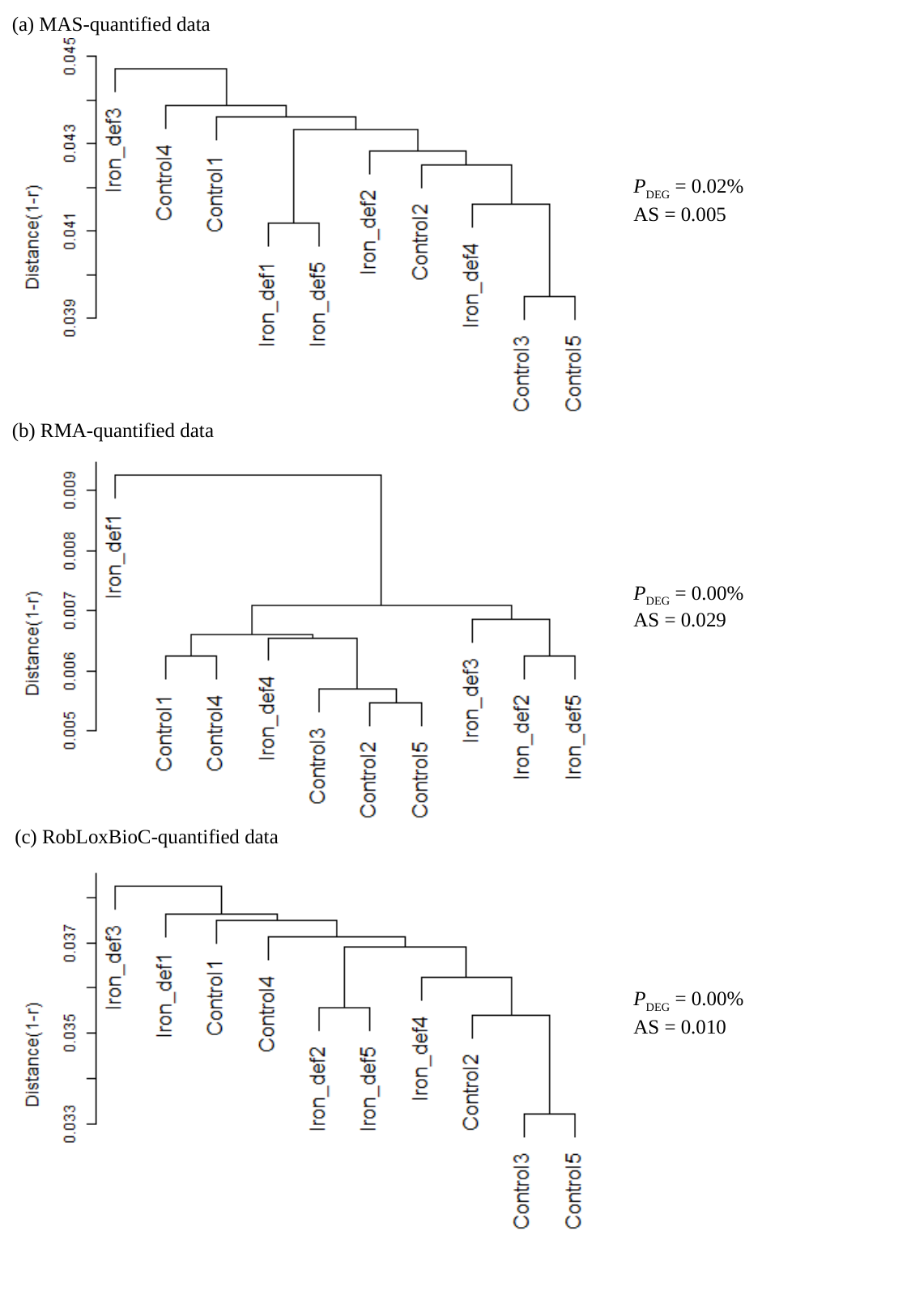

(a) MAS-quantified data
PDEG = 0.02%
AS = 0.005
(b) RMA-quantified data
PDEG = 0.00%
AS = 0.029
(c) RobLoxBioC-quantified data
PDEG = 0.00%
AS = 0.010
