# Supplementary figures and images for: Silhouette Scores for Arbitrary Defined Groups in Gene Expression Data and Insights into Differential Expression Results
Source: Biol Proced Online. 2018 Mar 1;20:5. doi: 10.1186/s12575-018-0067-8 (PMC5831220; doi:10.1186/s12575-018-0067-8)

## Slide 1
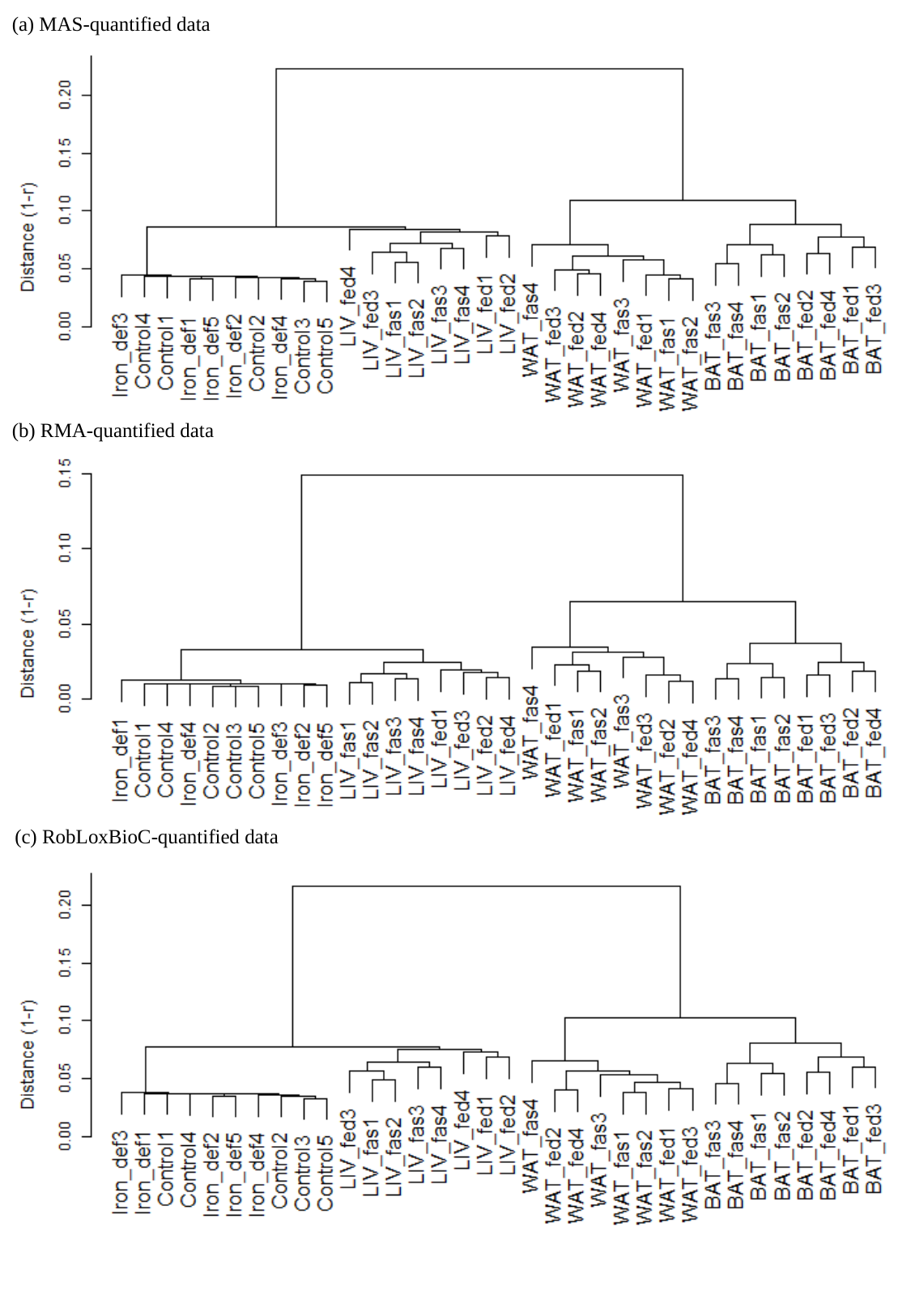

(a) MAS-quantified data
(b) RMA-quantified data
(c) RobLoxBioC-quantified data

Supplement: Supplementary file 8 — HSC dendrograms for merged microarray data (Nakai + Kamei). HSC dendrograms for (a) MAS-, (b) RMA-, and (c) RobLoxBioC-quantified data are shown. These data consist of 31,099 genes × 34 samples (24 from Nakai and 10 from Kamei data). (PPTX 62 kb) [file 12575_2018_67_MOESM8_ESM.pptx]
